# In vitro model of ischemic heart failure using human induced pluripotent stem cell–derived cardiomyocytes

**DOI:** 10.1172/jci.insight.134368

**Published:** 2021-05-24

**Authors:** Justin Davis, Ahmad Chouman, Jeffery Creech, Andre Monteiro da Rocha, Daniela Ponce-Balbuena, Eric N. Jimenez Vazquez, Ruthann Nichols, Andrey Lozhkin, Nageswara R. Madamanchi, Katherine F. Campbell, Todd J. Herron

**Affiliations:** 1 Frankel Cardiovascular Regeneration Core Laboratory, Department of Internal Medicine, Division of Cardiovascular Medicine,; 2 Center for Arrhythmia Research,; 3 Department of Internal Medicine, Division of Cardiovascular Medicine,; 4 Department of Biological Chemistry, and; 5 Department of Molecular and Integrative Physiology, University of Michigan, Ann Arbor, Michigan, USA.

**Keywords:** Cardiology, Cell Biology, Cardiovascular disease, Heart failure

## Abstract

Human induced pluripotent stem cell–derived cardiomyocytes (hiPSC-CMs) have been used extensively to model inherited heart diseases, but hiPSC-CM models of ischemic heart disease are lacking. Here, our objective was to generate an hiPSC-CM model of ischemic heart disease. To this end, hiPSCs were differentiated into functional hiPSC-CMs and then purified using either a simulated ischemia media or by using magnetic antibody-based purification targeting the nonmyocyte population for depletion from the cell population. Flow cytometry analysis confirmed that each purification approach generated hiPSC-CM cultures that had more than 94% cTnT^+^ cells. After purification, hiPSC-CMs were replated as confluent syncytial monolayers for electrophysiological phenotype analysis and protein expression by Western blotting. The phenotype of metabolic stress–selected hiPSC-CM monolayers recapitulated many of the functional and structural hallmarks of ischemic CMs, including elevated diastolic calcium, diminished calcium transient amplitude, prolonged action potential duration, depolarized resting membrane potential, hypersensitivity to chemotherapy-induced cardiotoxicity, depolarized mitochondrial membrane potential, depressed SERCA2a expression, reduced maximal oxygen consumption rate, and abnormal response to β_1_-adrenergic receptor stimulation. These findings indicate that metabolic selection of hiPSC-CMs generated cell populations with phenotype similar to what is well known to occur in the setting of ischemic heart failure and thus provide a opportunity for study of human ischemic heart disease.

## Graphical abstract

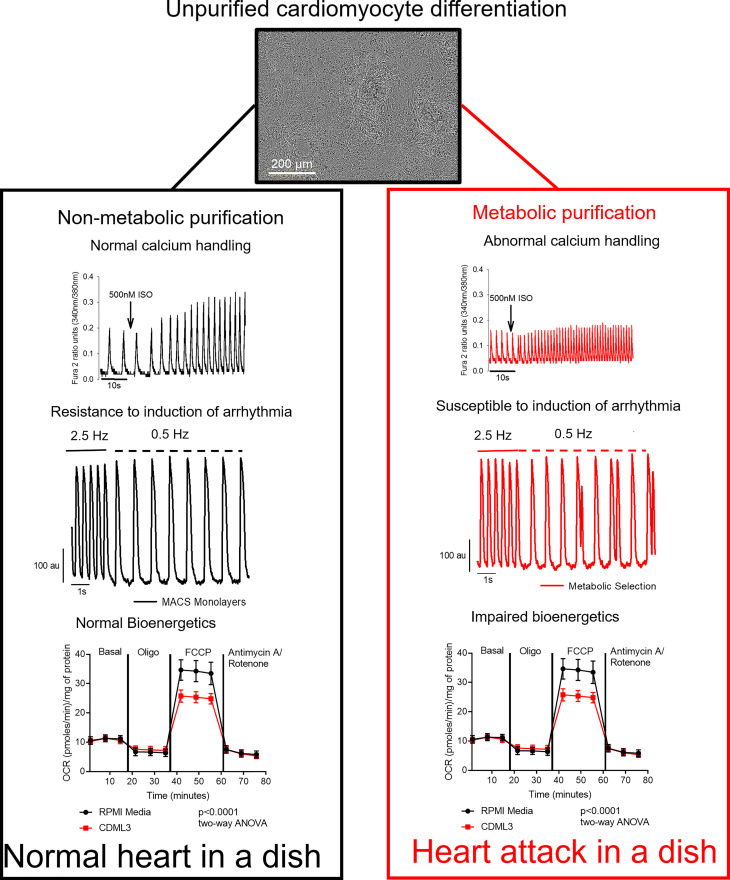

## Introduction

The use of human induced pluripotent stem cell–derived cardiomyocytes (hiPSC-CMs) now makes it possible to generate a virtually unlimited supply of human CMs that can be used for research purposes (1, 2). hiPSC-CMs have been utilized to model many monogenic cardiac diseases, such as long QT syndrome (3, 4), hypertrophic cardiomyopathy (5), catecholaminergic polymorphic ventricular tachycardia (6), and Timothy syndrome (7). On the other hand, there are few reports on the generation of hiPSC-CM models of acquired heart disease, such as ischemic heart disease and failure. Ischemic heart failure is a significant health problem that affects over 5 million patients in the United States and currently the only cure is cardiac transplantation. The development of new and effective therapies to treat ischemic heart failure will be hastened by the availability of an in vitro hiPSC-CM model of ischemic cardiomyopathy.

Importantly the in vitro hiPSC-CM ischemic heart failure model should recapitulate the molecular and functional hallmarks that are well known to occur in patients and research models of heart failure. For example, contractile function of the individual CMs is depressed in human heart failure and this has been confirmed in numerous animal models of ischemic heart failure (8). Molecular and genetic modification of key contractile and calcium-handling proteins underlie the poor contractile function of the failing heart and CMs. Specifically, heart failure is associated with reduced SERCA2a gene expression and reduced phospholamban (PLN) phosphorylation (9, 10). These proteins directly regulate the amount of calcium available to trigger contraction and therefore represent molecular targets for heart failure therapy. SERCA2a gene overexpression in the heart rapidly hastens the removal of cytosolic calcium during diastole, thus alleviating diastolic dysfunction. In fact, SERCA2a gene therapy was shown in vitro to improve the contractile function of human CMs isolated from patients afflicted with heart failure and is being tested as a new therapy for patients with heart failure (11–13). Thus, an in vitro model of human ischemic heart failure should present with reduced contraction, reduced calcium flux amplitudes, reduced SERCA2a protein expression, prolonged calcium transient duration (CaTD), prolonged action potential duration (APD), and reduced PLN phosphorylation. Additionally, the functional phenotype should be rescued using established SERCA2a gene therapy approaches known to improve CM contractile function. Further, the ischemic heart failure in vitro model should recapitulate elevated sensitivity to cardiotoxic therapies such as doxorubicin chemotherapy. Our goal here was to demonstrate these features of human heart failure phenotypes using hiPSC-CMs in vitro.

Currently, the most widely utilized approach for purification of hiPSC-CMs relies on marked metabolic differences in glucose and lactate metabolism between CMs and non-CMs (14). Tohyama et al. (14) cultured pluripotent stem cell–derived cells with glucose-depleted culture medium supplemented with abundant lactate and found that only CMs survived. This method of hiPSC-CM purification has been adopted by many laboratories and is a highly efficient method for large-scale purification (15, 16). However, this same media composition has been utilized historically to simulate ischemia preconditioning in cultured adult CMs (17). Also, a similar glucose-free solution has previously been referred to as “injury solution” to produce localized ischemic injury to cardiac monolayers (18, 19). Here we hypothesized that this simulated ischemia media (0 glucose, abundant lactate) generates purified hiPSC-CMs with an ischemic heart failure–like phenotype. To test this hypothesis, we compared the phenotype of metabolic stress–purified hiPSC-CMs with the phenotype of hiPSC-CMs purified using a commercially available magnetic-activated cell sorting (MACS) approach (Miltenyi Biotec). The MACS approach and subsequent enrichment of the hiPSC-CMs was recently described and validated for use in cardiotoxicity proarrhythmia assays (20, 21).

**Figure 1:**
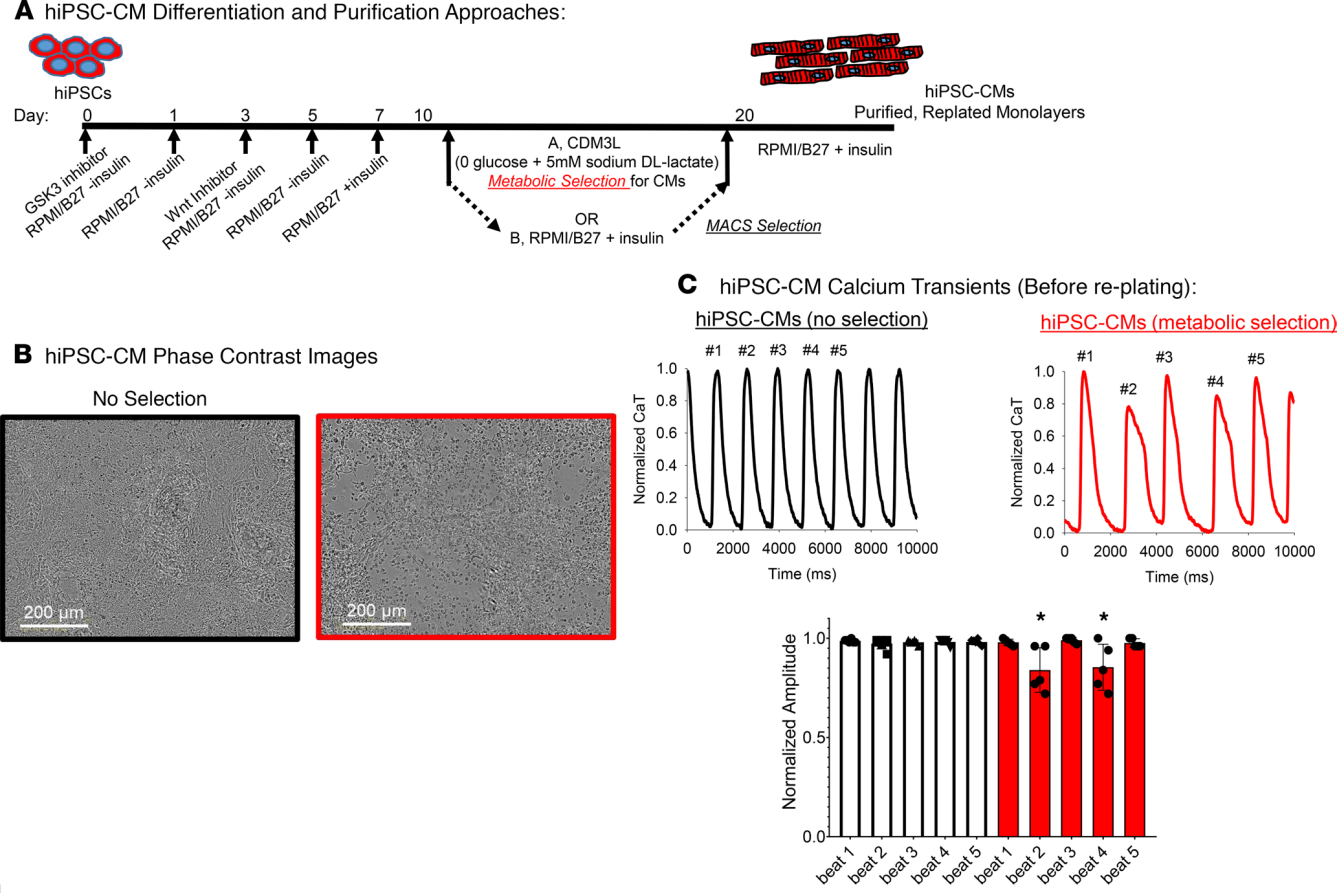
hiPSC-CM differentiation protocol and purification approaches. (**A**) Timeline of small molecule–based cardiac-directed differentiation and purification approaches. (**B**) Phase-contrast images showing the impact of metabolic selection on monolayer confluence (19-1-11 hiPSC line, day 24). (**C**) hiPSC-CM spontaneous calcium transient recordings in 19-9-11 hiPSC-CMs without any selection (black, *n* = 6 monolayers) and with metabolic selection (red, *n* = 5 monolayers). Spontaneous calcium transient alternans were induced by metabolic selection media (red traces and bars). *Denotes significant difference of amplitude between even and odd numbered beats. Quantification in **C** shows the relative amplitude of each beat after beat 1, normalized to beat 1 amplitude. Without selection, the amplitude did not vary beat to beat (beat 2 = 0.97 ± 0.02, beat 3 = 0.98 ± 0.01; *n* = 6). However, in lactate selection media (CDML3), the beat amplitude varied on a beat-to-beat basis (beat 2 = 0.84 ± 0.11, beat 3 = 0.99 ± 0.01; *n* = 6; paired *t* test, *P* < 0.05), indicating calcium transient alternans. hiPSC-CMs, human induced pluripotent stem cell–derived cardiomyocytes.

Figure 1A outlines the general cardiac-directed differentiation and purification approaches used here. All functional and structural phenotype analysis was performed on syncytial hiPSC-CM monolayers. The electrophysiological dysfunction (calcium transient alternans, red traces in Figure 1C) apparent during metabolic selection is depicted in Figure 1C and provided us rationale for further study.

## Results

hiPSC-CMs were enriched for preparation of syncytial monolayers with a metabolic selection protocol or with MACS targeting non-CMs for immunodepletion (Figure 1A). Two separately established hiPSC lines were used here: (a) 19-9-11 hiPSC line (WiCell, iPS DF19-9-11T.H) (22) and (b) BJ-hiPSC line (generated in house using mRNA reprogramming) (23, 24).

**Figure 2:**
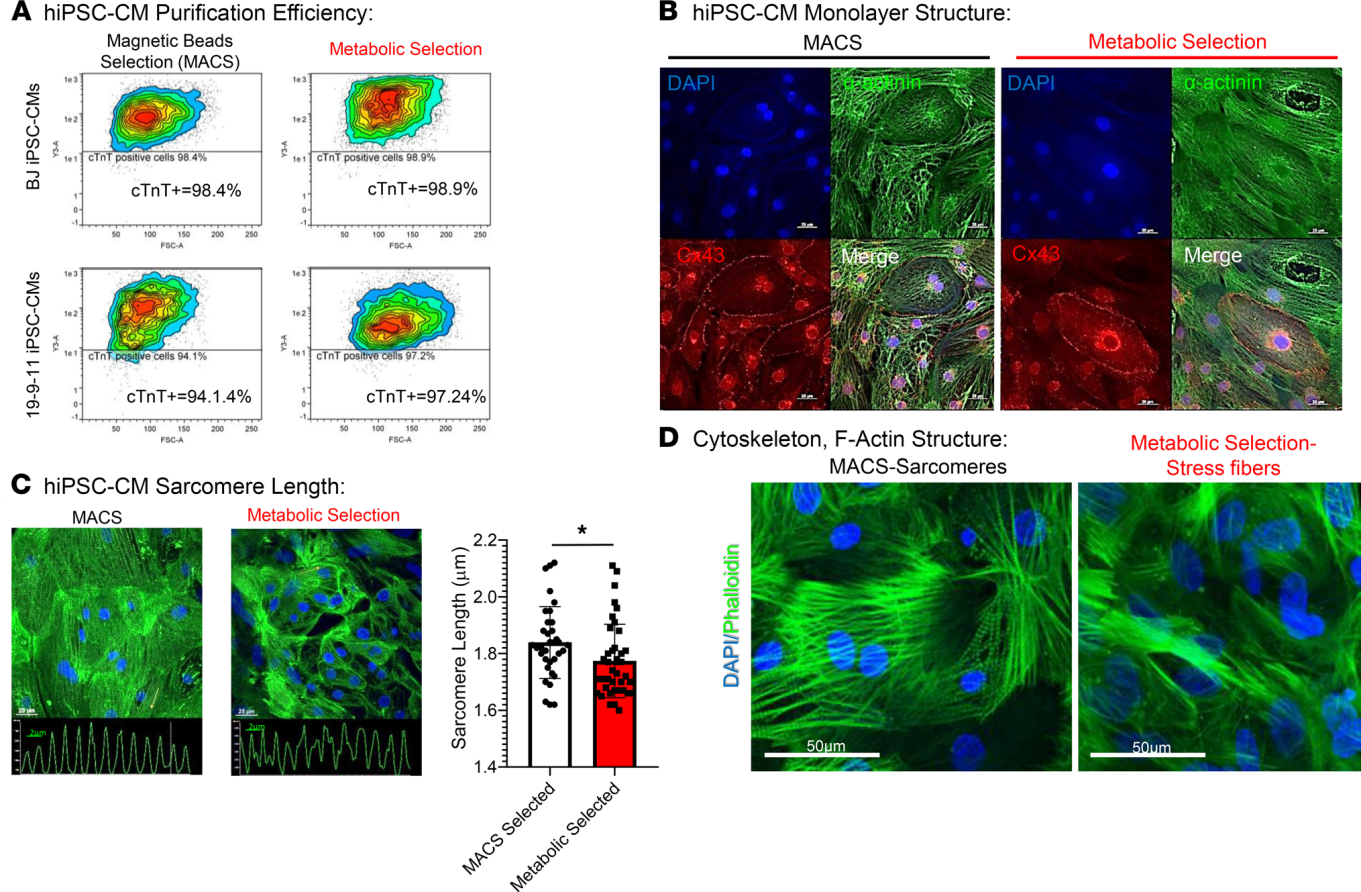
hiPSC-CMs generated using 2 distinct purification approaches. (**A**) Each purification approach is equally effective to enrich hiPSC-CM population analyzed by flow cytometry with cTnT labeling. (**B**) Connexin43 (Cx43) mislocalization was apparent in metabolic stress–selected monolayers. (**C**) Metabolic stress–selected hiPSC-CMs had shorter sarcomere length (MACS = 1.85 ± 0.17 μm; *n* = 35 vs. metabolic stress selection = 1.77 ± 0.13 μm; *n* = 40; unpaired *t* test, *P = 0.009). Sarcomere length was quantified using the repeating fluorescence pattern of the α-actinin staining. (**D**) Phalloidin staining was used to examine the actin cytoskeleton. Stress fibers rather than sarcomeres were identified in the metabolic stress–selected hiPSC-CMs. hiPSC-CMs, human induced pluripotent stem cell–derived cardiomyocytes; cTnT, cardiac troponin I; MACS, magnetic-activated cell sorting.

CM enrichment was successful with both methods across the cell lines used, and the 2 methods yielded similar percentages of CMs (Figure 2A, metabolic selection = 97.03 ± 2.9% purity, *n* = 4 and MACS selection = 95.2 ± 3.0% cTnT^+^ cells, *n* = 4). CMs selected with each of the 2 different methods formed functional syncytial cardiac monolayers with cell-cell propagation of electrical impulses as described before (21, 23–26); nevertheless, there were several phenotypic differences between CMs obtained by the 2 methods such as heterogeneous expression and mislocalization of connexin 43 (Figure 2B), decrease in sarcomere length and organization (Figure 2C), and the appearance of actin stress fibers in metabolic stress–selected hiPSC-CMs (Figure 2D and Supplemental Figures 3 and 4). Additionally, functional phenotyping of CMs indicated changes in important functional characteristics of CMs like intracellular calcium handling and electrophysiology.

### Metabolic selection alters intracellular calcium handling and blunts β-adrenergic response of CMs.

**Figure 3:**
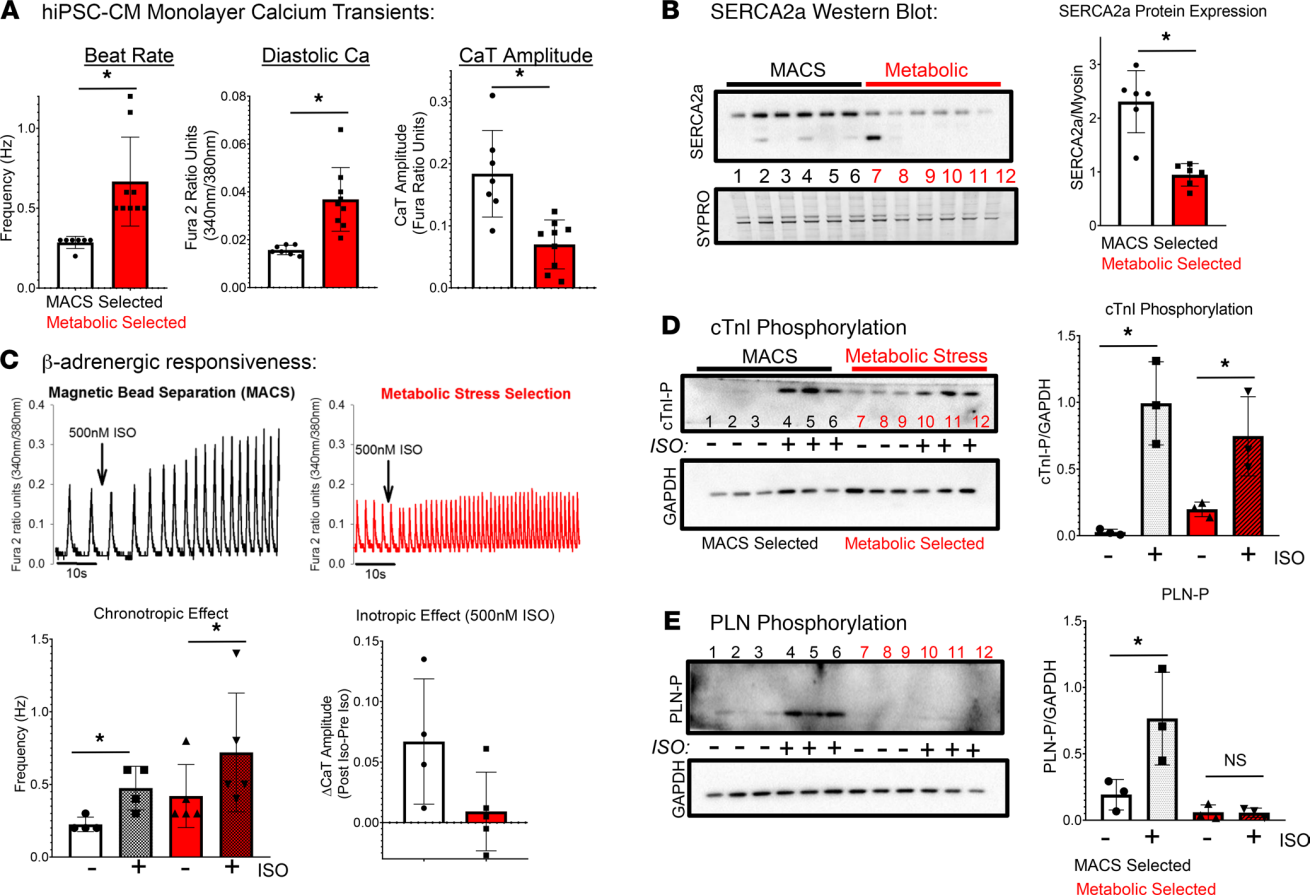
hiPSC-CM purification approach effects on CM intracellular calcium flux. (**A**) Metabolic stress selection significantly alters baseline calcium flux of hiPSC-CMs. (**B**) SERCA2a expression levels were reduced in metabolic stress–selected hiPSC-CMs. (**C**) MACS-purified CMs (black traces, symbols) responded as expected to isoproterenol; however, metabolic stress–selected hiPSC-CMs responded only with positive chronotropy. (**D**) cTnI phosphorylation state was elevated at baseline in metabolic stress–selected hiPSC-CMs (0.19 ± 0.05, *n* = 3 vs. 0.03 ± 0.02 AU, *n* = 3). cTnI phosphorylation increased after isoproterenol stimulation (metabolic stress = 0.75 ± 0.29, *n* = 3 and MACS = 0.99 ± 0.30 AU, *n* = 3). (**E**) PLN phosphorylation levels (PLN-P) were similarly low at baseline in each group, and only in MACS-sorted hiPSC-CMs was PLN-P detected at significant levels after isoproterenol treatment (metabolic stress = 0.05 ± 0.06 AU, *n* = 3 vs. MACS = 0.77 ± 0.35 AU, *n* = 3; 1-way ANOVA, *P = 0.02). hiPSC-CMs, human induced pluripotent stem cell–derived cardiomyocytes; cTnI, cardiac troponin I; PLN, phospholamban; MACS, magnetic-activated cell sorting.

Figure 1C shows that metabolic selection induced calcium transient alternans in hiPSC-CM monolayers prior to replating, whereas calcium transient alternans were not observed in nonselected hiPSC-CM monolayers. Next, hiPSC-CM monolayers selected with the 2 purification methods were loaded with calcium-sensitive dye (Fura2AM) for quantification of intracellular calcium transients and displayed divergent functional phenotypes. The frequency of spontaneous intracellular calcium activations was higher in syncytial monolayers of CMs prepared after metabolic selection (0.52 ± 0.07 Hz, *n* = 9 vs. MACS = 0.27 ± 0.05 Hz, *n* = 7; significant difference *t* test, *P* < 0.001; Figure 3A) compared with those prepared after MACS sorting. Furthermore, metabolic stress–selected hiPSC-CM monolayers had higher diastolic cytosolic calcium concentrations compared with MACS-enriched CMs (0.04 ± 0.008 fura ratio units, *n* = 15 vs. MACS = 0.01 ± 0.004, *n* = 13; *t* test, *P* < 0.001; Figure 3A). Calcium transient amplitude was significantly greater in the MACS-enriched hiPSC-CM monolayers compared with metabolic stress–selected monolayers (MACS = 0.14 ± 0.05 fura ratio units, *n* = 13 vs. metabolic selection = 0.09 ± 0.03 fura ratio units, *n* = 15; *t* test, *P* = 0.014; Figure 3A).

Next, we sought to determine the molecular mechanism to account for the differences in calcium flux observed between metabolic and MACS-based purification approaches. Western blotting was employed to determine the relative expression levels of SERCA2a, the primary calcium pump of CMs responsible for cytosolic calcium sequestration into the sarcoplasmic reticulum. Impaired intracellular calcium handling in metabolic stress–selected CMs was mechanistically associated with significantly lower expression of SERCA2a protein (0.94 ± 0.21 AU, *n* = 6 vs. 2.31 ± 0.56 AU, *n* = 6; significant difference *t* test, *P* = 0.002; Figure 3B) compared with MACS-selected CMs. Furthermore, in Figure 3C, imaging intracellular calcium flux (with Fura2) after isoproterenol treatment demonstrated that MACS-selected CMs present the expected chronotropic and inotropic responses to β-adrenergic stimulation, because both spontaneous frequency and calcium transient amplitude significantly increased after isoproterenol treatment (from 0.27 ± 0.05 Hz to 0.5 ± 0.1 Hz, *n* = 3 and amplitude increased from 0.09 ± 0.06 Fura2 ratio units to 0.15 ± 0.10 fura ratio units, *n* = 3). On the other hand, in Figure 3C, β-adrenergic stimulation of CMs purified with metabolic selection did not induce an inotropic effect, because spontaneous frequency significantly increased (from 0.5 ± 0.08 up to 1.0 ± 0.4 Hz, *n* = 3 red traces) without concomitant increase in intracellular calcium amplitude (preisoproterenol = 0.08 ± 0.05 Fura2 ratio units and postisoproterenol amplitude = 0.06 ± 0.06 Fura2 ratio units, *n* = 3).

To further explore molecular mechanisms at play, we examined the phosphorylation state of 2 key CM proteins after isoproterenol (500 nmol/L^-1^) stimulation of β_1_-adrenergic receptors, namely, cardiac troponin I (cTnI) and PLN. CMs harvested from syncytial monolayers prepared after metabolic selection had higher baseline cTnI phosphorylation than MACS-sorted CMs but presented lower cTnI phosphorylation after isoproterenol treatment (Figure 3D). Investigation of PLN phosphorylation levels by Western blotting provided complementary mechanistic insight on metabolic stress–selected CMs intracellular calcium dysregulation. Untreated metabolic stress–selected hiPSC-CM monolayers presented lower levels of phosphorylated PLN than MACS-selected CMs (Figure 3E). After isoproterenol treatment, MACS-selected CMs had an approximately 15-fold increase in phosphorylated PLN, whereas metabolic stress–selected CMs did not have any detectable increase in phosphorylated PLN.

### Metabolic stress selection impacted hiPSC-CM electrophysiology.

**Figure 4:**
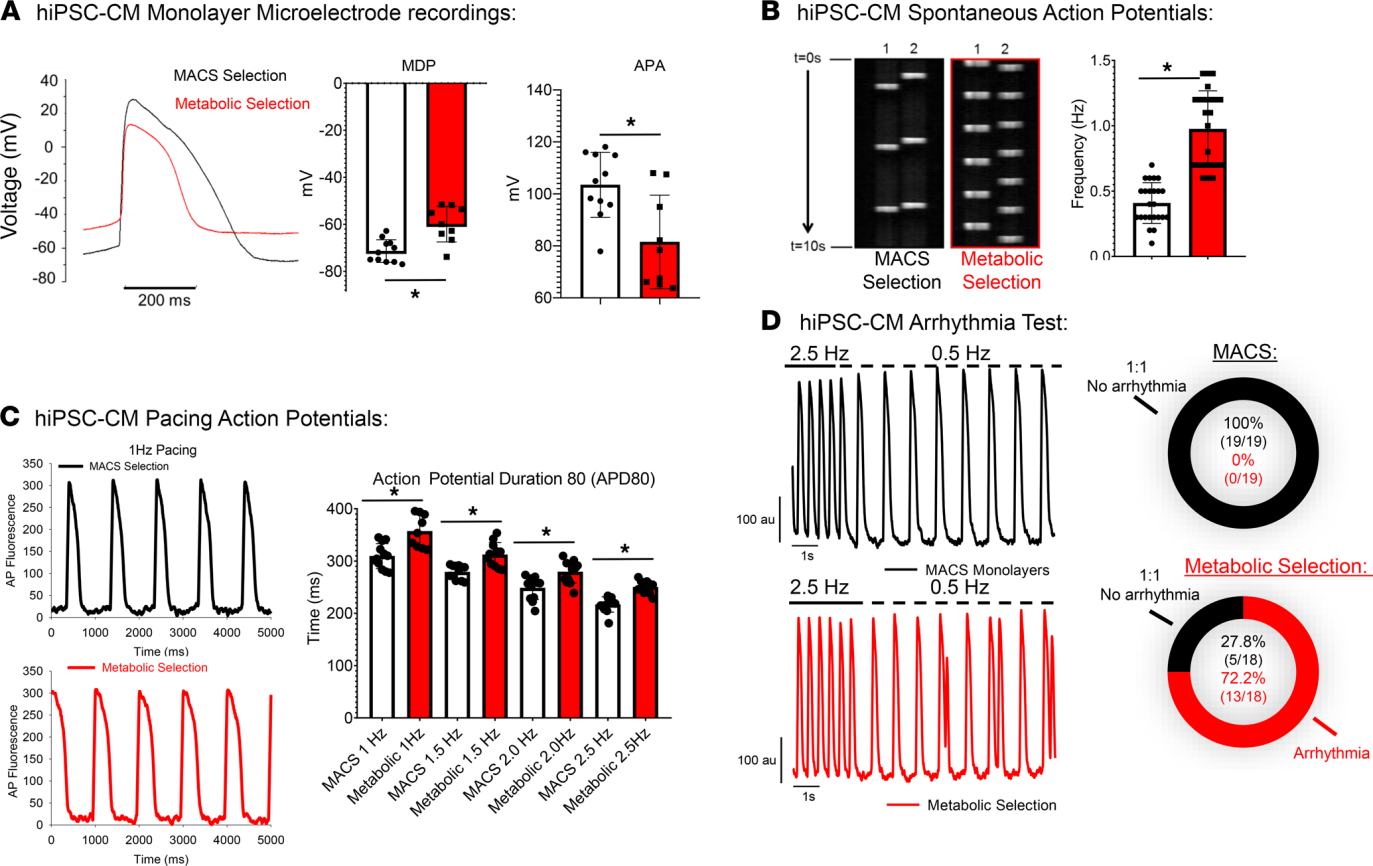
hiPSC-CM purification approach impacts electrophysiology phenotypes. (**A**) Microelectrode recordings show MDP was depolarized in metabolic stress–selected hiPSC-CMs APA was greater in MACS-selected hiPSC-CM monolayers (MACS = 103.5 ± 12.5 mV; *n* = 11 vs. metabolic stress selection = 81.5 ± 17.9 mV; *n* = 9; unpaired *t* test, *P < 0.001). (**B**) Metabolic stress–selected monolayers had significantly greater spontaneous beating frequency. (**C**) Metabolic stress selection monolayers had significantly longer APD80 than MACS-selected monolayers over a range of pacing frequencies (1 Hz: metabolic = 357.12 ± 6.85 ms; 1.5 Hz: metabolic = 312.24 ± 7.03 ms; 2.0 Hz: metabolic = 279.38 ± 6.21 ms; 2.5 Hz metabolic = 250.29 ± 4.20 ms; *n* = 9-12 monolayers) vs. (1 Hz: MACS = 309.56 ± 6.85 ms;1.5 Hz: MACS = 279.16 ± 3.83 ms; 2.0 Hz: MACS = 248.60 ± 6.31 ms; 2.5 Hz: MACS = 216.93 ± 4.20 ms; *n* = 12 monolayers, mean ± SEM). (**D**) Arrhythmia testing was done by abrupt slowing of pacing from 2.5 to 1.0 Hz with continuous recording. MACS-purified monolayers could follow the pacing change with 1:1 capture; however, 72.2% of metabolic stress–selected monolayers displayed arrhythmias. hiPSC-CM, human induced pluripotent stem cell–derived cardiomyocyte; MDP, maximal diastolic potential; APA, action potential amplitude; MACS, magnetic-activated cell sorting.

After hiPSC-CM purification using MACS or metabolic stress selection, cells were replated as electrically and mechanically confluent monolayers for electrophysiological analysis as described previously (20, 21, 27). First electrophysiology of hiPSC-CM monolayers was investigated using sharp microelectrode recordings or optical mapping with a voltage sensitive dye. Figure 4A shows original action potential recordings and quantification of maximal diastolic potential (MDP) and action potential amplitude (APA). MDP was less negative (depolarized) in metabolic stress–selected monolayers than MACS-purified hiPSC-CM monolayers (MACS = –71.4 ± 4.84 mV; *n* = 11 vs. metabolic stress hiPSC-CMs = –59.9 ± 7.59 mV, *n* = 9 significance, unpaired *t* test, *P* < 0.001). This baseline membrane potential difference translated to greater frequency of spontaneous activations in metabolic stress–selected monolayers (MACS = 0.41 ± 0.16 Hz; *n* = 24 monolayers vs. metabolic selection = 0.97 ± 0.29 Hz; *n* = 24 significant difference unpaired *t* test, *P* < 0.001) and lower action potential amplitude (APA) (Figure 4B). To precisely investigate APD, hiPSC-CM syncytial monolayers were paced at different frequencies during optical mapping with voltage sensitive dye (Figure 4C). Action potential duration (APD) at 80% (APD80) of repolarization was significantly longer in metabolic stress–selected hiPSC-CM monolayers than MACS-selected monolayers at 1.0, 1.5, 2.0, and 2.5 Hz pacing frequencies (Figure 4C). Finally, metabolic stress–selected and MACS-selected hiPSC-CM syncytial monolayers were submitted to an arrhythmia test consisting of abrupt decrease in pacing from 2.5 Hz to 1.0 Hz while recording optical action potentials. All syncytia prepared with MACS-selected CMs were able to follow the decrease in pacing frequency with 1:1 capture; however, 72% of metabolic stress–selected hiPSC-CM monolayers displayed early afterdepolarizations (Figure 4D).

### Effect of hiPSC-CM purification approach on mitochondrial membrane potential.

**Figure 5:**
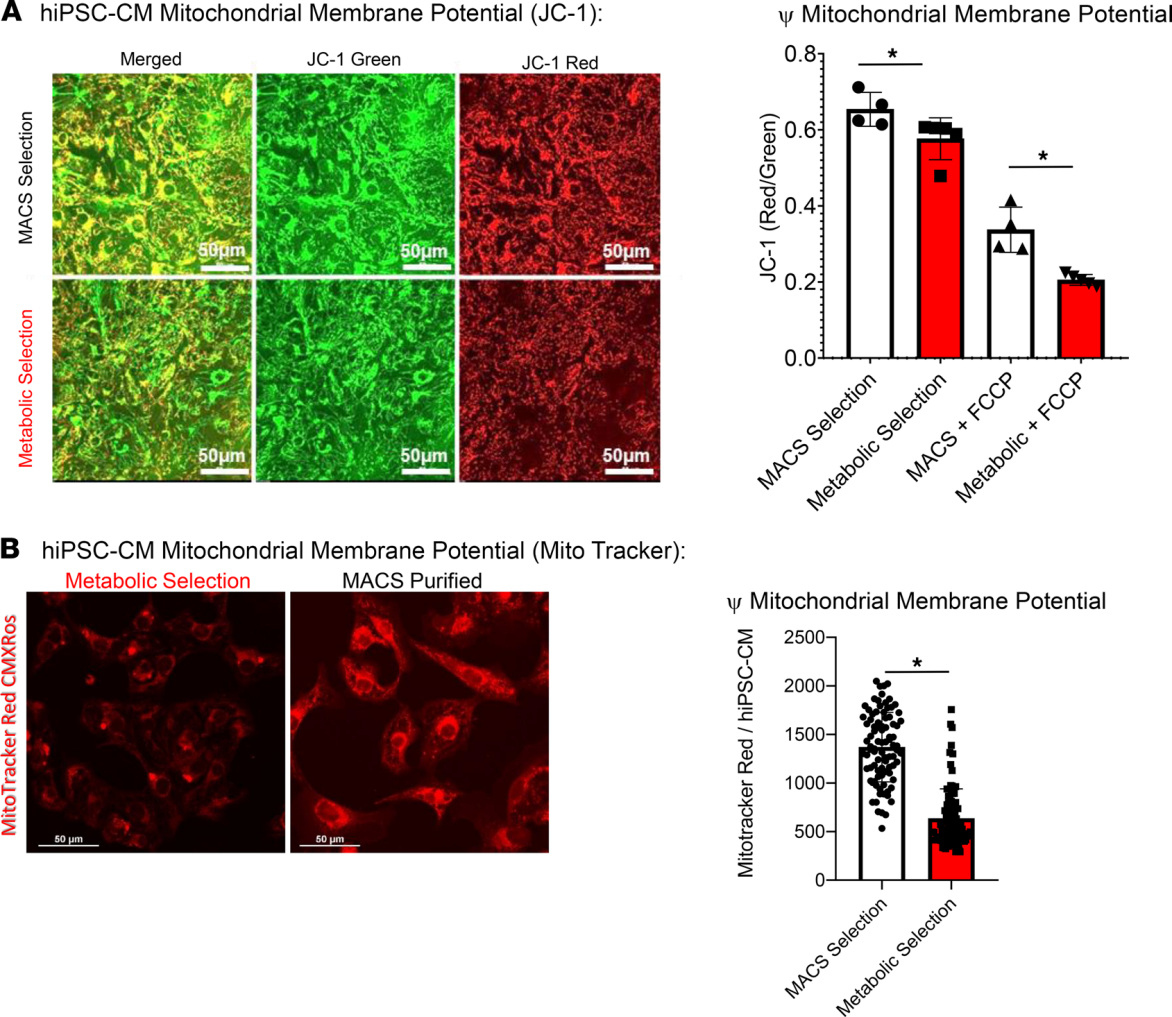
Metabolic selection induces mitochondrial dysfunction in hiPSC-CM monolayers. (**A**) JC-1 staining indicates more polarized mitochondria in MACS-purified monolayers. Numeric values are in the main text. (**B**) MitoTracker Red CMX Ros staining supports the JC-1 results. MitoTracker signal, MACS = 1369.11 ± 355.66 AU vs. metabolic stress selection = 635.45 ± 302.12, *t* test, *P < 0.001. hiPSC-CM, human induced pluripotent stem cell–derived cardiomyocyte; MACS, magnetic-activated cell sorting.

Mitochondria have been implicated in several human diseases including cardiac disease and are sensitive to energy substrates (28). Next, we determined the effect of the hiPSC-CM purification approach on mitochondrial membrane potential (Δψ_m_) using the carbocyanine compound, JC-1. Figure 5A shows confocal images of hiPSC-CM monolayers made from metabolic stress–selected or MACS-selected CMs loaded with JC-1. It is apparent in this image that there is a greater amount of red staining in the MACS-sorted CMs relative to metabolic stress–selected hiPSC-CMs. Indeed, quantification of the ratio of red/green fluorescence shows that metabolic stress–selected hiPSC-CMs have a lower ratio and thus have depolarized Δψ_m_ (0.57 ± 0.06 AU, *n* = 5 vs. MACS = 0.65 ± 0.04, *n* = 4; significant difference unpaired *t* test, *P* < 0.001). Δψ_m_ depolarization is indicated by the reduction of the red to green fluorescence intensity ratio and this difference persisted even after treatment with the mitochondrial uncoupler, FCCP (metabolic stress–selected hiPSC-CM = 0.21 ± 0.01 AU, *n* = 5 vs. MACS = 0.34 ± 0.06, *n* = 4). Further complementary analysis of mitochondrial membrane potential was done using MitoTracker Red CMXRos dye (Figure 5B). MitoTracker Red CMXRos dye accumulates in the mitochondria and its accumulation is dependent on mitochondrial membrane potential (29). Figure 5B shows greater MitoTracker Red dye accumulation in MACS-purified hiPSC-CMs, indicating more hyperpolarized, active mitochondria compared with CMs purified using metabolic stress selection.

### Metabolic selection increased susceptibility of CMs to doxorubicin-induced cardiotoxicity.

**Figure 6:**
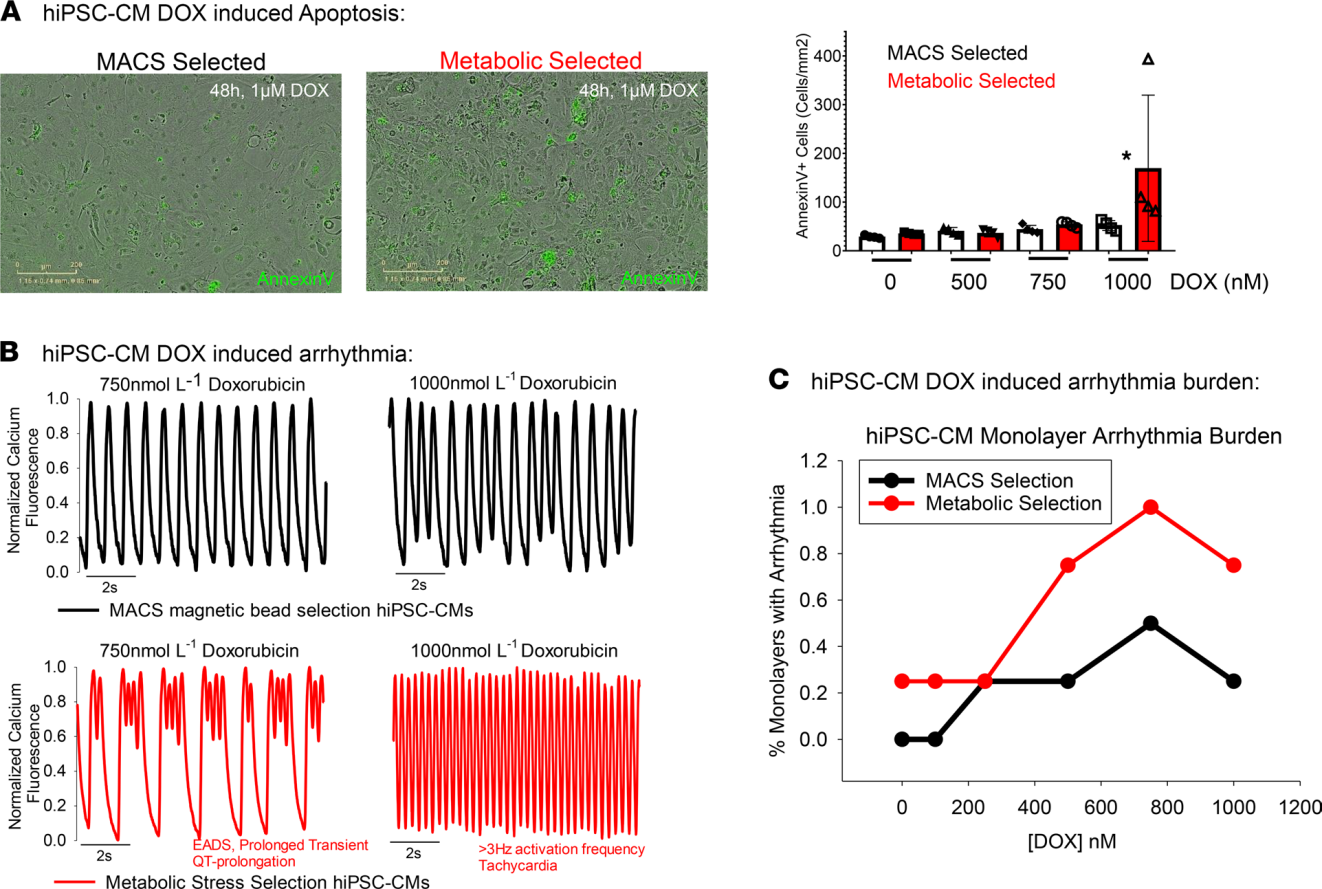
Metabolic stress–selected hiPSC-CM monolayers have increased sensitivity to chemotherapy-induced cardiotoxicity (DOX-TOX). (**A**) Annexin V staining indicates greater extent of apoptosis in metabolic-purified hiPSC-CMs treated with 1000 nM doxorubicin (DOX). (**B**) Calcium transient optical mapping using rhod2 example traces show greater sensitivity of electrophysiological function of metabolic stress selection hiPSC-CMs (red traces). (**C**) Metabolic stress–selected hiPSC-CM monolayers show arrhythmias in response to DOX-TOX with greater sensitivity than MACS-purified hiPSC-CM monolayers. hiPSC-CMs, human induced pluripotent stem cell–derived cardiomyocytes. hiPSC-CM, human induced pluripotent stem cell–derived cardiomyocyte; MACS, magnetic-activated cell sorting.

Doxorubicin (Adriamycin) is a chemotherapy drug with well-documented cardiotoxicity, referred to as DOX-TOX. DOX-TOX has been linked to direct effects on mitochondrial function and intracellular calcium flux. We hypothesized that metabolic stress–selected hiPSC-CMs are more sensitive to DOX-TOX, owing to the baseline differences in mitochondrial membrane potential, electrophysiology, and intracellular calcium flux identified in Figure 3, Figure 4, and Figure 5. To test this, metabolic stress– selected and MACS-selected CM monolayers were challenged with doxorubicin concentrations ranging from 100 nM to 1000 nM. First, cell survival was assessed in CM monolayers using annexin V staining (Figure 6A). Annexin V live-cell staining demonstrated similar DOX-TOX of metabolic stress–selected and MACS-selected CMs to doses equal to or below 750 nM of doxorubicin. However, the number of cells undergoing apoptosis after exposure to 1000 nM of doxorubicin was approximately 3 times higher in metabolic stress–selected CM syncytial monolayers than MACS-selected monolayers (169.4 ± 75.1 annexin V + cells/mm^2^, *n* = 4 vs. 51.8 ± 5.1 annexin V + cells/mm^2^, *n* = 4; mean ± sem, *t* test *P* < 0.001; Figure 6A).

Optical mapping with calcium-sensitive dye (rhod2AM; Figure 6, B and C) was performed after chronic exposure (48 h) to doxorubicin and the percentage of monolayers with arrhythmias was calculated for each group. Arrhythmia phenotypes included early after depolarization (EAD), extra calcium release, and tachycardia. Doxorubicin concentration of 250 nM or below did not induce arrhythmias in monolayers from either group. Doxorubicin concentrations of 500 nM or greater induced arrhythmias and significantly higher arrhythmia burden in metabolic stress–selected CMs syncytia than MACS-selected syncytial monolayers (Figure 6, B and C). Arrhythmia burden reached 100% in metabolic stress–selected hiPSC-CM syncytial monolayers at 750 nM of doxorubicin treatment with absolute predominance of EADs (Figure 6, B and C) and severe tachyarrhythmia at 1000 nM of doxorubicin.

### SERCA2a overexpression corrected CaTD prolongation associated with heart failure.

**Figure 7:**
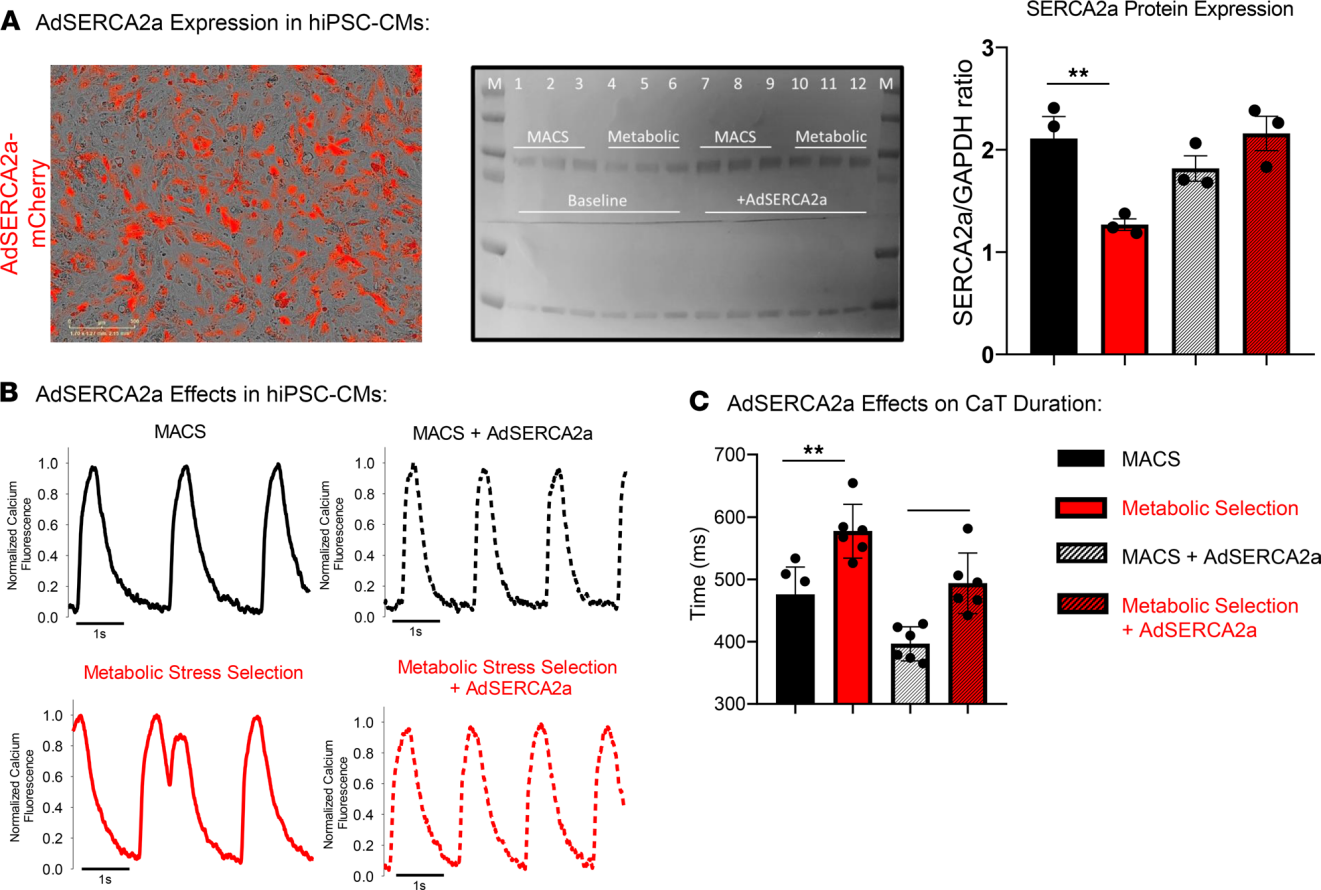
SERCA2a calcium pump gene therapy reverses hiPSC-CM heart failure phenotype. (**A**) Viral transduction was verified by mCherry live-cell expression in hiPSC-CM monolayers. Further, SERCA2a protein expression was determined by Western Blotting. Quantification indicates that SERCA2a protein expression was reduced in metabolic stress–treated hiPSC-CMs at baseline (MACS = 2.11 ± 0.22, *n* = 3 vs. metabolic stress = 1.27 ± 0.06, *n* = 3). AdSERCA2a treatment restored SERCA2a levels to control levels (metabolic stress plus Ad SERCA2a = 2.15 ± 0.17, *n* = 3; ANOVA, *P < 0.05). (**B**) Calcium transient measurements indicate that metabolic stress selection media prolongs the cardiac action potential and spontaneous arrhythmias were observed in the metabolic stress selection purification approach. hiPSC-CM monolayers treated with AdSERCA2a did not develop arrhythmias. (**C**) CaTD50 was prolonged in metabolic stress media–treated cells at baseline and was corrected to control values with AdSERCA2a gene therapy (CaTD50s: MACS baseline = 475.5 ± 18.1 ms; metabolic stress baseline = 577.37 ± 17.7 ms; MACS plus AdSERCA2a = 396.6 ± 11.2 ms; metabolic stress plus Ad SERCA2a = 493.67 ± 19.9 ms; ANOVA, **P = 0.002, ns). CaTD, calcium transient duration; hiPSC-CMs, human induced pluripotent stem cell–derived cardiomyocytes; MACS, magnetic-activated cell sorting.

Finally, we tested the effectiveness of SERCA2a gene therapy to improve the function of the metabolic stress–selected hiPSC-CMs. Recombinant adenovirus (Vector Biolabs) designed to deliver SERCA2a gene and mCherry promoter was applied to MACS-sorted and metabolic stress–treated monolayers (100 MOI). mCherry expression indicated robust expression throughout the monolayers (Figure 7A) and Western blotting confirmed elevated SERCA2a expression levels (Figure 7A). Figure 7B shows calcium transients recorded in each condition. AdSERCA2a overexpression shortened the calcium transient duration significantly. Functional analysis measuring intracellular calcium flux (fluo-4) indicated spontaneous arrhythmias in the metabolic stress media–treated cells and prolonged CaTD50. The quantification in Figure 7C indicates correction of the CaTD50 with SERCA2a gene therapy to control levels.

### Metabolic stress media affected the electrophysiological phenotypes of MACS-purified hiPSC-CMs and antibiotic resistance–purified hiPSC-CMs.

**Figure 8:**
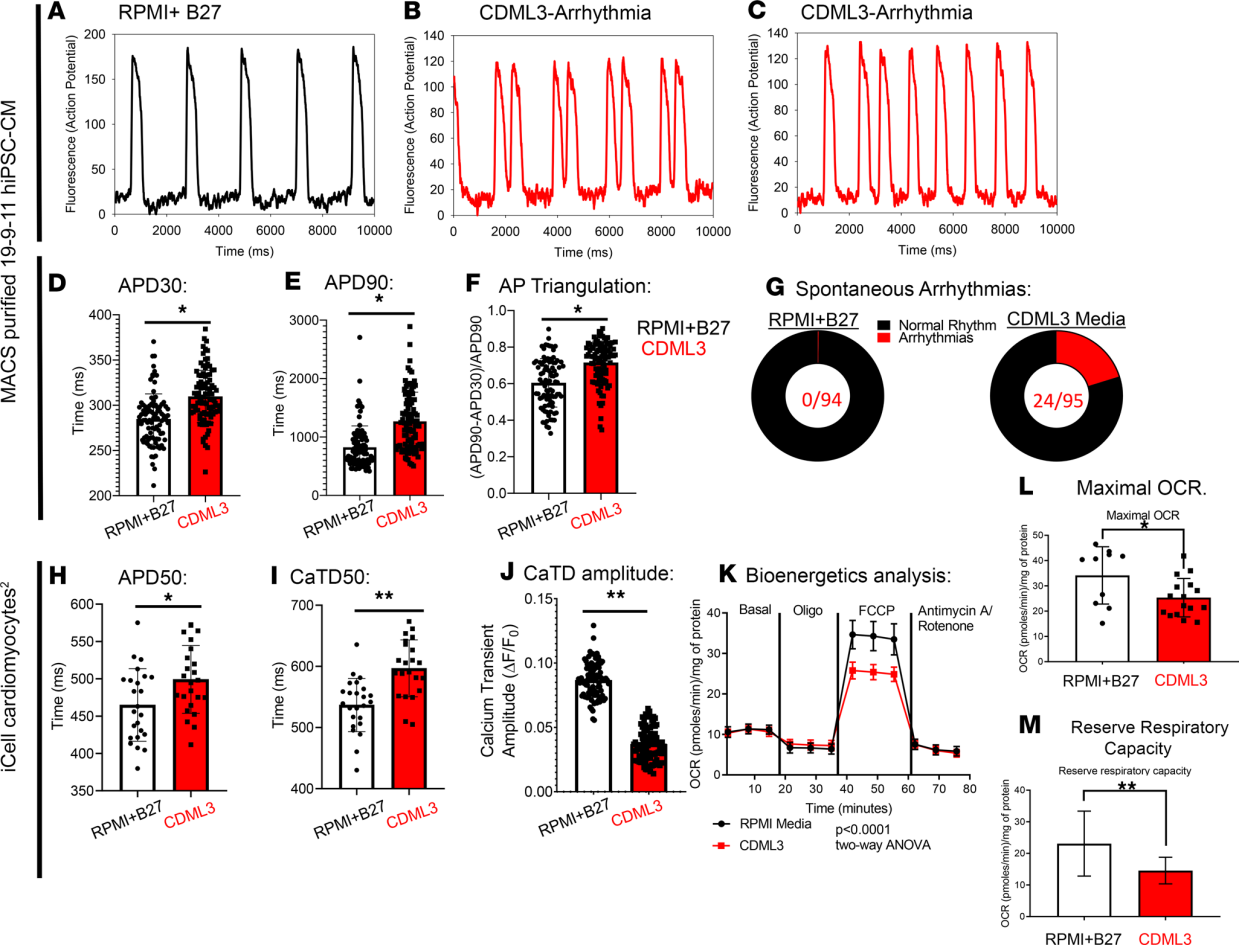
CDML3 metabolic selection media induces arrhythmia phenotypes in MACS-purified 19-9-11 hiPSC-CM monolayers and antibiotic resistance–purified hiPSC-CMs. (**A**) Action potential traces (fluovolt) of hiPSC-CMs maintained in RPMI plus B27 maintenance media. (**B** and **C**) CDML3 induced arrhythmia phenotypes observed using voltage sensitive dye. (**D**) APD30, (**E**) APD50, and (**F**) action potential triangulation are greater in CDML3-treated hiPSC-CMs. RPMI plus B27, APD30 = 284.7 ± 27.8 ms; CDML3 APD30 = 309.7 ± 29.0 ms; unpaired *t* tests, *P < 0.001, *n* = 94 and *n* = 95. RPMI APD90 = 823.2 ± 366.1 ms; CDML3 APD90 = 1266.7 ± 499.9 ms; unpaired *t* test, *P < 0.001. RPMI triangulation = 0.60 ± 0.13; CDML3 triangulation = 0.72 ± 0.12; unpaired *t* test, *P < 0.001. (**G**) None of the 94 wells showed spontaneous arrhythmias in RPMI plus B27 monolayers, whereas 24 of 95 monolayers presented with spontaneous arrhythmias in the CDML3-treated group. (**H–J**) Electrophysiology and calcium flux of iCell^2^ CMs is affected by maintenance in CDML3 media. APD50 and CaTD50 are prolonged in the CDML3 media–treated monolayers. Calcium transient amplitude of CDML3 media–treated monolayers plated in 96-well plates is reduced compared with RPMI-B27 maintenance media. (**K**) Seahorse measurement of OCR shows reduced maximal OCR (**L**) and reduced reserve respiratory capacity (**M**) in CDML3-treated hiPSC-CM monolayers. hiPSC-CMs, human induced pluripotent stem cell–derived cardiomyocytes; MACS, magnetic-activated cell sorting; OCR, oxygen consumption rate. Unpaired *t* test, **P < 0.05.

Next, we tested the effect of the CDML3 metabolic selection media on hiPSC-CMs that were purified using the MACS approach. This enabled testing the effect of the metabolic stress condition of 0 glucose and abundant lactate on hiPSC-CM function without interference of non-CMs death. Here we used the same hiPSC line (19-9-11), differentiated into CMs as outlined in Figure 1, and purified hiPSC-CMs using MACS selection. hiPSC-CMs were replated into 2 separate 96-well plates: 1 plate was cultured in maintenance media (RPMI/B27 plus insulin) and the other plate was cultured in CDML3 media, each for 10 days prior to electrophysiological recordings. Figure 8 outlines the effects of CDML3 media on hiPSC-CM electrophysiological function, assessed using voltage sensitive dye (fluovolt). Figure 8A shows representative action potential traces from hiPSC-CMs cultured in media containing glucose (RPMI plus B27). Figure 8, B and C, show examples of spontaneous arrhythmias recorded in hiPSC-CMs that were cultured in 0 glucose/abundant lactate media (CDML3) for 10 days after MACS sorting and replating. Importantly, all recordings were made in the same physiological salt solution (HBSS) after the media treatments, indicating that the effects of each media persist after media changes. Quantification of APD and action potential triangulation are presented in Figure 8, D–F. APD30 was significantly prolonged in hiPSC-CM monolayers cultured in CDML3 (309.7 ± 29.0 ms, *n* = 95 vs. 284.7 ± 27.8, *n* = 94; unpaired *t* test, *P* < 0.0001). APD90 was also significantly prolonged in hiPSC-CM monolayers cultured in CDML3 media (1266.7 ± 499.9 ms, *n* = 95 vs. 823.2 ± 366.1 ms, *n* = 94; unpaired *t* test, *P* < 0.0001). Action potential triangulation is a unitless parameter calculated by the following equation: action potential triangulation = (*APD*90 – *APD*30)/*APD*90, where *APD*90 refers to the amount of time that repolarization takes to return to 90% of the resting membrane potential and *APD*30 refers to the amount of time that repolarization takes to return to 30% of the resting membrane potential. The mathematical relationship between early (*APD*30) and late (*APD*90) repolarization is used as an index to predict arrhythmogenesis. Increased action potential triangulation occurs when the APD is prolonged and is used as an index to describe the potential to cause arrhythmias. hiPSC-CM monolayers cultured for 10 days in CDML3 media had greater action potential triangulation values (0.72 ± 0.12 AU, *n* = 95 vs. 0.60 ± 0.13 AU, *n* = 94; unpaired *t* test, *P* < 0.0001). Increased action potential triangulation increases the odds for observing spontaneous arrhythmias in these monolayers cultured in 0 glucose and abundant lactate (CDML3). Indeed, Figure 8G shows that the increased APD values and increased action potential triangulation manifested as a greater number of spontaneous arrhythmias occurring in the CDML3-treated group (22 of 95 wells with arrhythmias vs. 00 of 94 wells with arrhythmias).

To rigorously test the effect of CDML3 media on a separate hiPSC-CM source, high-purity, commercially available CMs (iCell cardiomyocytes^2^) were cultured in CDML3 media for 10 days. APD50% was significantly increased by metabolic stress induced with CDML3 (APD50%: 499.24 ± 9.3 ms, *n* = 24; *P* = 0.01) compared with RPMI media (APD50%: 464.9 ± 9.91 ms, *n* = 24; Figure 8H). Intracellular CaTD50% was significantly increased in syncytia treated with CDML3 (596.77 ± 9.48 ms, *n* = 24) compared with syncytia cultured in control media (536.82 ± 8.89 ms, *n* = 24; *P* < 0.0001; Figure 8I). Further, in 2 separate 96-well plates, in which 1 full plate was maintained in CDML3 media and the other plate in RPMI media, we found reduced intracellular calcium amplitude induced by the CDML3 media treatment (RPMI B27 = 0.0866 ± 0.0013 AU, *n* = 96 and CDML3 = 0.0369 ± 0.0012 AU, *n* = 96, *P* < 0.0001; Figure 8J). These data collectively support the hypothesis that media containing 0 glucose and abundant lactate can be used to simulate cardiac injury and create an arrhythmogenic substrate in syncytia of commercially available hiPSC-CMs.

At last, in another set of experiments, MACS-purified hiPSC-CMs were cultured in CDML3 or RPMI media prior to bioenergetics assessment with Seahorse XFe96 analyzer (19-9-11 hiPSC-CMs 7 days of CDML3 exposure). Oxygen consumption rate was affected by 7 days of exposure of MACS separated CMs to CDML3 media. Mitochondrial oxygen consumption rate (OCR) was significantly lower in CMs after 7 days of exposure to metabolic stress, although there were no differences in ATP-driven and nonmitochondrial OCR (Figure 8, K–M). Bioenergetics analysis showed significant reduction of maximal respiration (RPMI B27 = 34.14 ± 3.58 [pmoles/min]/mg of protein and CDML3 = 25.34 ± 1.9 [pmoles/min]/mg of protein, *P* = 0.02; Figure 8L) and decrease in respiratory reserve capacity (RPMI B27 = 23.04 ± 3.25 [pmoles/min]/mg of protein and CDML3 = 14.56 ± 1.05 [pmoles/min]/mg of protein, *P* = 0.006; Figure 8M) in CMs cultured under metabolic stress conditions. Seahorse XFe96 analyzer data concatenate with mitochondrial membrane potential data as described in the previous section.

## Discussion

To date, the most widely used method for hiPSC-CM purification relies on metabolism differences between CMs and non-CMs. The widely used hiPSC-CM purification media contains 0 glucose and abundant lactate, a media composition that has been used historically to simulate ischemia in native adult CMs in vitro (17–19). Here, we tested the hypothesis that this metabolic-mediated hiPSC-CM selection subjects the CMs to a metabolic stress that induces an ischemic-like phenotype. To this end, we completed a direct comparison of the functional and structural phenotypes of hiPSC-CMs purified with either metabolic selection media (0 glucose, abundant lactate) or MACS using commercially available reagents (Figure 1). A robust feature of this approach is that we were able to differentiate between a batch of hiPSCs and hiPSC-CMs and then utilize either purification approach on the same batch of cells. Thus, the comparisons are tightly time-matched.

We report distinct structural and functional phenotypes induced by each hiPSC-CM purification approach. hiPSC-CM selected by means of alteration of energetic substrate presented a reduction in membrane-bound connexin43 in contrast to hiPSC-CMs that were not submitted to metabolic stress (Figure 2B). Changes in connexin43 expression are not pathognomonic of cardiac ischemia; nevertheless, it has been long known from studies using animal models of ischemia/hypoxia and postmortem human specimens that ischemia induces alterations in connexin43 expression, localization, and phosphorylation; impacts cardiac function; and produces a substrate for reentrant arrhythmias (30–35).

CMs exposed to 0 glucose and abundant lactate had sarcomere shortening in comparison with hiPSC-CMs selected with MACS (Figure 2C); this was similar to the decrease in sarcomere length demonstrated previously in in vitro and animal models of myocardial infarction, hypoxia, and hypoxia with glycolytic blockade (36–38). Similar sarcomere disorganization was apparent with cTnT staining in a separate hiPSC cell line (Supplemental Figure 1; supplemental material available online with this article; https://doi.org/10.1172/jci.insight.134368DS1). Furthermore, staining with phalloidin revealed the abundant presence of nonstriated F-actin fibers among sarcomeres (also known as stress fibers) in metabolic stress–selected cells (Figure 2D), whereas MACS-purified hiPSC-CMs presented striated pattern of phalloidin staining characteristic of sarcomeres. Stress fibers are present in different cell types and have important roles in cell adhesion and migration. In CMs, stress fibers participate in early stages of sarcomere morphogenesis (39) and they can be observed in dedifferentiated CMs adjacent to immobilized areas of the heart (40). Whether the presence of stress fibers in CMs submitted to metabolic stress is indicative of dedifferentiation or immature phenotype is out of the scope of this manuscript. Nevertheless, CMs purified with glucose deprivation and high availability of lactate present nuclear expression of Ki67 and retain proliferation potential (Supplemental Figure 2; ref. 14), which suggest the hypothesis that metabolic selection delays hiPSC-CM maturation. Although we have not further explored the expression of genes associated to maturation of CMs, Ordono et al. (41) has shown that the exposure of hiPSC-CMs to 6 mM of lactate for 3 days is able to induce upregulation of genes associated to dedifferentiation and proliferation.

Functionally, metabolic stress–selected hiPSC-CM monolayers showed electrophysiological and intracellular calcium-handling properties reminiscent of CMs isolated from ischemic failing hearts. Figure 3 summarizes the differences in hiPSC-CM calcium handling between the 2 purification approaches. Metabolic stress–selected syncytial monolayers had higher diastolic calcium concentrations, reduced calcium transient amplitudes, and reduced SERCA2a expression levels at baseline relative to MACS-sorted hiPSC-CMs (Figure 3). These are all features reminiscent of the phenotypes well documented for heart failure CMs.

The expression and phosphorylation status of calcium-handling proteins regulate cardiac function and are implicated as causal for poor function of the failing heart. SERCA2a expression, for example, is significantly reduced in animal models of heart failure and this molecular defect contributes to diastolic dysfunction and poor contraction of the failing heart (10). In fact, increasing SERCA2a expression levels has shown to be effective to restore cardiac function in animal models of heart failure, and clinical trials have been conducted to deliver the SERCA2a gene to patients with heart failure (42). Here we have discovered that metabolic stress selection of hiPSC-CMs produces CMs with reduced SERCA2a expression compared with hiPSC-CMs purified using the MACS approach (Figure 3B). Furthermore, we used SERCA2a gene therapy approach in vitro to restore the function and arrhythmia phenotypes of metabolic stress–selected hiPSC-CM monolayers (Figure 7).

Another calcium-handling protein consistently implicated in contributing to heart failure phenotypes is PLN (43). It is well established that PLN is phosphorylated upon myocardial β-adrenergic stimulation and this contributes to positive inotropic and lusitropic effects in healthy hearts. In cases of heart failure, however, phosphorylation of PLN is reduced and this is attributed to dysfunctional adrenergic signaling (9, 43, 44). Here we have found that metabolic selection generated hiPSC-CMs in which PLN phosphorylation did not occur upon isoproterenol treatment (Figure 3E). MACS-selected hiPSC-CM monolayers, however, showed significant phosphorylation of PLN upon isoproterenol stimulation (Figure 3E). This suggests a dysfunctional or underdeveloped β-adrenergic signaling pathway in metabolic stress–selected hiPSC-CMs. These molecular differences most likely underlie the disparate functional responses to isoproterenol presented in Figure 3C. MACS-selected hiPSC-CM monolayers responded to isoproterenol with positive chronotropic and inotropic responses. On the other hand, metabolic stress–selected hiPSC-CM monolayers showed only a positive chronotropic effect of isoproterenol treatment with no significant positive inotropic effect. cTnI phosphorylation, however, was induced by isoproterenol treatment in both groups. These molecular and cellular differences of intracellular calcium flux in hiPSC-CMs purified by distinct methods resemble the extensive comparisons that have been made historically between native CMs isolated from healthy and failing hearts.

Key electrophysiological parameters including MDP, APA, and rate adaptation were also impacted by purification approach. Figure 4 outlines the key electrophysiological differences that we observed between metabolic stress–selected hiPSC-CMs and MACS-selected hiPSC-CMs. Microelectrode recordings in Figure 4A show differences in MDP and APA between hiPSC-CMs purified by each method. MDP was significantly depolarized in metabolic stress–selected hiPSC-CMs syncytial monolayers compared with MACS-selected hiPSC-CMs. Depolarization of MDP and smaller APA may be attributed to elevated diastolic calcium concentrations observed in metabolic stress–selected hiPSC-CMs relative to MACS-selected hiPSC-CM monolayers. Depolarization of MDP also contributed to the differences of baseline spontaneous contraction rate between the groups (Figure 4B); depolarized MDP set the membrane potential closer to threshold for firing an action potential. APD was significantly prolonged in metabolic stress–selected hiPSC-CM monolayers even over a range of electrical pacing frequencies (Figure 4C). APD prolongation is a feature well known to occur in heart failure, that contributes to QT prolongation of the EKG, and that increases risk for suffering a fatal arrhythmia in patients with heart failure (45). Arrhythmia testing by rapid reduction of pacing frequency from 2.5 to 0.5 Hz revealed significantly greater incidence of EADs and extra contractions in metabolic stress–selected hiPSC-CM monolayers relative to MACS-selected monolayers (Figure 4D). The electrophysiological differences between hiPSC-CM monolayers purified by each method outlined in Figure 1 resemble the well-established differences between healthy and heart failure CMs (45).

Furthermore, we have observed changes in mitochondrial function that recapitulate bioenergetic features of CMs submitted to ischemia (Figure 5 and Figure 8, K–M), namely, an overall reduction of mitochondrial membrane potential and function. This was measured using fluorescent mitochondrial membrane potential sensitive dyes and with a respirometry assay performed with a Seahorse XFe96 analyzer. Figure 8, K–M, show the bioenergetic differences between hiPSC-CM monolayers that were maintained in either CDML3 (metabolic selection media) or glucose containing RPMI media for 7 days. Metabolic selection media produced hiPSC-CM monolayers with reduced maximal OCR and respiratory reserve capacity. The reduction of maximal OCR is similar to clinical data showing that human CMs isolated from the left atrial appendage from patients suffering chronic myocardial ischemia have loss of maximal OCR (46). Inherent mitochondrial functional differences also underlie the enhanced sensitivity to DOX-TOX observed in metabolic stress–selected hiPSC-CMs (Figure 6). In combination with elevated calcium concentrations in metabolic stress–selected hiPSC-CMs, the mitochondrial dysfunction combines to increase sensitivity to DOX-TOX. Metabolic stress–selected hiPSC-CMs showed increased apoptosis after doxorubicin treatment compared with MACS-sorted hiPSC-CMs (Figure 6B). Also, DOX-TOX indexed by arrhythmia occurrence was more apparent in metabolic stress–selected hiPSC-CMs relative to MACS-sorted hiPSC-CM monolayers (Figure 6, C and D). This indicates that the purification approach should be carefully considered when using hiPSC-CMs to predict patient-specific sensitivity to chemotherapy-induced cardiotoxicity.

The approach of metabolically starving nonmyocytes to enrich CM populations derived from stem cells has been employed for over 7 years (14) and has proven to be an effective approach. The metabolic starvation technique is attractive, owing to its ease of use, applicability to bulk processing, and extremely low cost. Here we have used the same metabolic starvation approach with subtle modification for comparison with a extracellular receptor magnetic bead–based approach that is commercially available (Miltenyi Biotec). Compared with the MACS-based purification, metabolic selection generates CMs with a heart failure–like molecular and functional phenotype. This provides a acquired heart failure model system for in vitro testing and research. There are some slight differences in protocols that require discussion. The first reports of using metabolic starvation used 8 days of treatment for purification of cells, and later publications using this method applied longer durations up to 10 days (16). Figure 1 shows that we used 10 days of metabolic media selection to obtain hiPSC-CMs for our comparisons, except for OCR experiments that were performed after 7 days of continuous exposure to CDM3L. Although we demonstrated that 7 days of treatment with CDM3L affects energy metabolism of CMs, we cannot state that shorter exposures to CDM3L such as used by Sharma et al. (47) induce an ischemic phenotype and this should be subjected to further investigation. During the differentiation protocol, previous reports (14–16) began the metabolic media purification treatment on day 10; however, we have initiated the metabolic selection on day 14. The timing of introducing metabolic selection and the duration of exposure may affect cellular phenotypes. Despite the subtle protocol differences, the rigorous experimental design using time matched controls in each differentiation experiment for each purification approach here enabled the comparisons between hiPSC-CM purification approaches.

### Conclusion.

Heart failure affects approximately 6.5 million Americans (48), the majority of whom suffer ischemic heart failure, which generally occurs secondary to coronary heart disease. Although current heart failure treatments are palliative in nature and cardiac transplantation is possible, they are inadequate to address a significant portion of the population. A major obstacle to the development of new and effective heart failure treatments has been the lack of viable human cardiac tissue and myocytes available for research. Our results indicate that human heart failure molecular and functional phenotypes can be modeled in vitro using metabolic selection of hiPSC-CMs. The use of acquired heart failure in vitro models using human cardiac syncytial monolayers offers advantages over the use of animal models and provides a testing platform for the development of new heart failure therapies. The use of syncytial monolayers of human CMs can be accomplished only by using PSC-CMs and here we have validated that these monolayers are sensitive to metabolic stress and can recapitulate many molecular and functional features well known to occur in CMs obtained from failing human hearts.

## Methods

### hiPSC maintenance.

Vector free hiPSCs were maintained on matrigel-coated 6-well plates using Xeno-free media (iPS Brew, Miltenyi Biotec) essentially as previously described (20, 23). Two vector-free control cell lines were utilized to determine efficiency of CM purification approaches (19-9-11 and BJ iPSCs). The 19-9-11 hiPSC line was obtained from WiCell and the BJ iPSC line was generated from commercially available human dermal fibroblasts in the laboratory using mRNA reprogramming. hiPSCs were maintained as colonies and passaged once every week. Pluripotent stem cell use was approved by the University of Michigan HPSCRO Committee.

### Cardiac-directed differentiation and hiPSC-CM purification.

Figure 1A outlines the cardiac-directed differentiation and purification protocols. We utilized the highly efficient small-molecule approach relying on temporal modulation of the Wnt signaling pathway (49). On day 14, cardiac differentiation cultures were switched to metabolic stress selection media (CDM3L, 0 glucose, 5 mM sodium DL-lactate) or maintained in CM maintenance media (RPMI/B27 plus insulin). Phase-contrast images of the differentiated CMs in CDM3L or RPMI maintenance media are shown in Figure 1B and indicate significant nonmyocyte cell death in CDM3L media, as expected based on previous reports (16). Figure 1C data show time-matched spontaneous calcium transient recordings of hiPSC-CM monolayers either with no selection or on day 10 of metabolic stress selection process prior to replating. The nonmyocyte cell death creates holes in the monolayer that, in addition to the high-lactate concentrations, contribute to the observed dysfunction. For metabolic selection of hiPSC-CMs, cell cultures were kept in CDM3L for 10 days and on day 24 purified hiPSC-CMs were trypsinized and replated as confluent monolayers for phenotype analysis. This is a slight deviation from the original reports of Tohyama et al., which used 6 to 8 days of metabolic stress selection with CDM3L to purify stem cell–derived CMs to 93% and 98% respectively (14, 50, 51). There is however, a precedence for our use of 10 days of CDM3L metabolic stress selection media in the work of Burridge et al. (16). In 2014, Burridge et al. (16) reported that 6–10 days of glucose deprivation using CDM3L was optimal to purify CMs from 85% to >95% purity. In fact, 10 days of selection was found to purify hiPSC-CMs to a greater extent than shorter time periods (6 days). For MACS hiPSC-CM purification, differentiation plates were kept in normal maintenance media and processed for CM purification on day 24 of the protocol (Miltenyi Biotec, PSC-Derived Cardiomyocyte Isolation Kit, Human). The MACS purification approach uses antibody to target a nonmyocyte extracellular epitope to magnetize the nonmyocytes, which are trapped in a magnetic field while nonlabeled CMs flow through the magnetic field and are collected, similarly to the alternative approach to the use of SIRPA antibodies for positive selection of CMs suggested by Dubois et al. (52). In each group the media was changed every other day. Flow cytometry was performed to assess the relative purity of CM cultures purified with the 2 methods by probing cTnT expression (Anti-cardiac Troponin T-APC; Miltenyi Biotec, 130-120-403). On the day of MACS purification, hiPSC-CMs were replated in parallel to the metabolic stress–selected hiPSC-CMs for phenotype comparison. Plating procedures have been described recently in detail (20, 23, 26). In each condition, phenotype analysis to study the structure and function of the hiPSC-CM monolayers was done 7 days after re plating. This approach enabled phenotype analysis of the same batch of hiPSC-CMs that were time matched but purified by distinct methods.

Experiments were done to determine the effectiveness of an established gene therapy reported to reverse heart failure phenotypes (these data are in Figure 8). Ad-mCherry-hATP2A2 was obtained from Vector Biolabs, stored at –80C, and applied to hiPSC-CM monolayers plated in 96-well plates using MOI = 100 (50,000 hiPSC-CMs per well). mCherry expression was detected using live-cell time-lapse imaging (Incucyte Zoom, Essen Bioscience) and expression was robust by 48 h after gene transfer. After optical mapping of intracellular calcium flux using fluo4AM (10 μM), monolayers were collected/solubilized in SDS sample buffer for Western blot detection of SERCA2a protein.

### Cardiac monolayer optical mapping and electrophysiology.

hiPSC-CM monolayer electrophysiology was measured using optical mapping or microelectrode recording as previously described (20, 23, 26). Action potential properties were measured using the voltage sensitive dye, fluovolt (Thermo Fisher), or by microelectrode recording. APD80 restitution was quantified using field stimulation of monolayers (20V, 5 ms duration, various Hz). Arrhythmia screening was done using a dynamic stimulation frequency assay as previously described (53). Briefly, monolayers were paced at 2.5 Hz and action potential responses to immediate slowing of pacing frequency (1.0 Hz) were quantified.

Calcium transients were recorded using rhod2AM, fluo-4AM, or the more quantitative calcium dye, Fura2AM. For Fura2AM measurements, monolayers were replated on to glass bottom dishes for microscopic imaging using an IonOptix system with alternating excitation between 340 nm and 380 nm ultraviolet light with emission recordings in the green spectrum (515 nm). Fura2 measurements are independent of cellular motion and amount of dye loaded and thus reflect intracellular calcium concentration changes. Fura2 measurements are expressed as ratio units. In each purification approach, intracellular calcium response to β-adrenergic receptor stimulation (500 nM isoproterenol) was quantified using Fura2-loaded monolayers. Rhod2-loaded monolayers (10 μM) were used to determine sensitivity to doxorubicin-induced arrhythmias in large monolayers.

### Control experiments using commercially available purified hiPSC-CMs.

In control experiments, we tested the hypothesis that CDML3 metabolic stress media also induces electrophysiological abnormalities in commercially available human iPSC-CMs. To test the effect of CDML3 metabolic stress media on hiPSC-CMs from another source, we applied this media to commercially available human CMs (iCell2, Cellular Dynamics International) that are purified by mechanisms other than those used in this study. These cells were handled, plated, and used for optical mapping as previously outlined (20, 21). First, we generated a 96-well plate of these cells, using 50,000 cells per well to form monolayers. One half of the plate was cultured in RPMI (glucose containing) media and the other half of the plate was cultured in CDML3 (0 glucose, high lactate) media. After 10 days in culture, the plate was used for functional analysis with half of the plate being used for action potential measurements (Fluovolt) and the other half being used for calcium transient analysis (fluo-4AM). Next, to determine the effect of CDML3 media on calcium transient amplitudes, 2 additional 96-well plates of iCell^2^ CMs were generated. In one plate, all 96 wells were cultured in RPMI media for 10 days and the other plate was maintained in CDML3 media for the same 10 days. On day 10, cells were loaded with rhod2AM (10 μM for 15 min) and spontaneous calcium flux was recorded using the LED-based optical mapping system.

### Mitochondrial membrane potential measurements.

Mitochondrial membrane potential (Δψ_m_) was determined using the carbocyanine compound JC-1, the optimal dye to use for quantification of changes of ψ in CMs (54, 55). JC-1 dye exhibits potential dependent accumulation in mitochondria, indicated by a shift in fluorescence emission from green to red. As a result, mitochondrial depolarization is indicated by a decrease of the red/green fluorescence intensity ratio. JC-1 dye was loaded into CMs (2 μM, 30 min) in the incubator at 37°C. Red and green emission was recorded in live cells using a Nikon A1R confocal microscope with the appropriate laser and detection wavelengths. FCCP (50 μM, carbonilcyanide p-trifluoromethoxyphenylhydrazone), a potent uncoupler of mitochondrial oxidative phosphorylation, was applied to validate the JC-1 measurements.

### Western blotting analysis.

Protein expression was analyzed by Western blotting essentially as previously described (20, 23). For each purification approach, SERCA2a protein expression was determined using a monoclonal antibody (Thermo Fisher, MA3-919; 1:300). Phospho-specific antibodies were used to determine phosphorylation levels of cTnI (Cell Signaling Technology, 4004S; 1:100) and PLN (Millipore, 07-052; 1:150) with or without isoproterenol treatment (500 nM).

### Immunofluorescence.

Monolayer structure was observed by immunofluorescence as previously described (14). For each purification approach, sarcomeric α-actinin (MilliporeSigma, A7811; 1:300) and connexin 43 (MilliporeSigma, C6219; 1:100) protein expression were visualized using monoclonal antibodies. Cell nuclei were also labeled using DAPI (1:1000). α-Actinin spacing was measured using fluorescence intensity-space plots as shown in Figure 2C. Confocal images were collected using a Nikon A1R confocal microscope (×60 objective) from 5 separate monolayers generated using each purification method. Average sarcomere spacing was quantified from at least 7 intensity-space plots drawn perpendicular to separate sarcomere patterns for each image.

### Apoptosis assay.

The extent of hiPSC-CM apoptosis immediately after each purification approach was quantified using Annexin V Green reagent (Essen Bioscience, catalog 4642). Monolayers resulting from each purification method were plated on PDMS and incubated with annexin V (Thermo Fisher, A13201) and doses of doxorubicin ranging from 0 nM to 1000 nM for 48 hours. Cells were then imaged using the Incucyte Zoom time-lapse microscope (Essen Biosciences) and annexin V fluorescence analyzed with its software.

### Statistics.

Unpaired 2-tailed *t* tests were used to compare statistics between 2 groups. One-way ANOVA was used to compare means of 3 or more groups. *P* < 0.05 was considered significant.

### Study approval.

Experiments using human iPSC lines were approved by the Human Pluripotent Stem Cell Research Oversight Committee (HPSCRO) of the University of Michigan. iPSC lines were obtained from commercial sources. Original material was obtained with informed consent.

## Author contributions

Experimental design was by all authors. Cell culture experiments, including stem cell maintenance, cardiac differentiation, purification, and replating, were done by JD, AC, JC, AMDR, and KFC. Microelectrode recordings of action potentials were done by DPB and ENJV. Optical mapping of action potentials and calcium transients were done by JD, TJH, JC, RN, and AMDR. Mitochondrial membrane potentials were measured by JD, TJH, and AMDR. AL, AMDR, and NRM executed bioenergetic analysis using the SeaHorse. All authors contributed to writing and editing the manuscript.

## Supplementary Material

Supplemental data

## Version 1 04/20/2021

In-Press Preview

## Version 2 05/24/2021

Electronic publication

## References

[1] Kamp TJ, Lyons GE (2009). On the road to iPS cell cardiovascular applications. Circ Res.

[2] Zhang J (2009). Functional cardiomyocytes derived from human induced pluripotent stem cells. Circ Res.

[3] Kamp TJ (2011). An electrifying iPSC disease model: long QT syndrome type 2 and heart cells in a dish. Cell Stem Cell.

[4] Itzhaki I (2011). Modelling the long QT syndrome with induced pluripotent stem cells. Nature.

[5] Lan F (2013). Abnormal calcium handling properties underlie familial hypertrophic cardiomyopathy pathology in patient-specific induced pluripotent stem cells. Cell Stem Cell.

[6] Novak A (2015). Functional abnormalities in iPSC-derived cardiomyocytes generated from CPVT1 and CPVT2 patients carrying ryanodine or calsequestrin mutations. J Cell Mol Med.

[7] Yazawa M (2011). Using induced pluripotent stem cells to investigate cardiac phenotypes in Timothy syndrome. Nature.

[8] Herron TJ (2010). Ca2+-independent positive molecular inotropy for failing rabbit and human cardiac muscle by alpha-myosin motor gene transfer. FASEB J.

[9] Chu G, Kranias EG (2006). Phospholamban as a therapeutic modality in heart failure. Novartis Found Symp.

[10] Gianni D (2005). SERCA2a in heart failure: role and therapeutic prospects. J Bioenerg Biomembr.

[11] del Monte F (1999). Restoration of contractile function in isolated cardiomyocytes from failing human hearts by gene transfer of SERCA2a. Circulation.

[12] Park WJ, Oh JG (2013). SERCA2a: a prime target for modulation of cardiac contractility during heart failure. BMB Rep.

[13] Zhai Y (2018). New insights into SERCA2a gene therapy in heart failure: pay attention to the negative effects of B-type natriuretic peptides. J Med Genet.

[14] Tohyama S (2013). Distinct metabolic flow enables large-scale purification of mouse and human pluripotent stem cell-derived cardiomyocytes. Cell Stem Cell.

[15] Burridge PW (2016). Human induced pluripotent stem cell-derived cardiomyocytes recapitulate the predilection of breast cancer patients to doxorubicin-induced cardiotoxicity. Nat Med.

[16] Burridge PW (2014). Chemically defined and small molecule-based generation of human cardiomyocytes. Nat Methods.

[17] Diaz RJ, Wilson GJ (2006). Studying ischemic preconditioning in isolated cardiomyocyte models. Cardiovasc Res.

[18] Vanden Hoek TL (1996). Reperfusion injury on cardiac myocytes after simulated ischemia. Am J Physiol.

[19] Arutunyan A (2001). Localized injury in cardiomyocyte network: a new experimental model of ischemia-reperfusion arrhythmias. Am J Physiol Heart Circ Physiol.

[20] da Rocha AM (2017). hiPSC-CM monolayer maturation state determines drug responsiveness in high throughput pro-arrhythmia screen. Sci Rep.

[21] da Rocha AM (2020). Detection of drug-induced torsades de pointes arrhythmia mechanisms using hiPSC-CM syncytial monolayers in a high-throughput screening voltage sensitive dye assay. Toxicol Sci.

[22] Yu J (2009). Human induced pluripotent stem cells free of vector and transgene sequences. Science.

[23] Herron TJ (2016). Extracellular matrix-mediated maturation of human pluripotent stem cell-derived cardiac monolayer structure and electrophysiological function. Circ Arrhythm Electrophysiol.

[24] Bizy A (2013). Myosin light chain 2-based selection of human iPSC-derived early ventricular cardiac myocytes. Stem Cell Res.

[25] Lee P (2012). Simultaneous voltage and calcium mapping of genetically purified human induced pluripotent stem cell-derived cardiac myocyte monolayers. Circ Res.

[26] Monteiro da Rocha A (2016). Deficient cMyBP-C protein expression during cardiomyocyte differentiation underlies human hypertrophic cardiomyopathy cellular phenotypes in disease specific human ES cell derived cardiomyocytes. J Mol Cell Cardiol.

[27] Zhang J (2019). Functional cardiac fibroblasts derived from human pluripotent stem cells via second heart field progenitors. Nat Commun.

[28] Rosca MG, Hoppel CL (2013). Mitochondrial dysfunction in heart failure. Heart Fail Rev.

[29] Zheng X (2016). Cardiomyocytes display low mitochondrial priming and are highly resistant toward cytotoxic T-cell killing. Eur J Immunol.

[30] Peters NS (1997). Disturbed connexin43 gap junction distribution correlates with the location of reentrant circuits in the epicardial border zone of healing canine infarcts that cause ventricular tachycardia. Circulation.

[31] Peters NS (1995). Myocardial gap junction organization in ischemia and infarction. Microsc Res Tech.

[32] Lampe PD (2006). Analysis of Connexin43 phosphorylated at S325, S328 and S330 in normoxic and ischemic heart. J Cell Sci.

[33] Kawamoto O (2014). Immunohistochemistry of connexin43 and zonula occludens-1 in the myocardium as markers of early ischemia in autopsy material. Histol Histopathol.

[34] Martins-Marques T (2015). Interacting network of the Gap Junction (GJ) Protein Connexin43 (Cx43) is modulated by ischemia and reperfusion in the heart. Mol Cell Proteomics.

[35] Zeevi-Levin N (2005). Gap junctional remodeling by hypoxia in cultured neonatal rat ventricular myocytes. Cardiovasc Res.

[36] Bing OH, Fishbein MC (1979). Mechanical and structural correlates of contracture induced by metabolic blockade in cardiac muscle from the rat. Circ Res.

[37] Schwartz P (1984). Ultrastructure of cultured adult myocardial cells during anoxia and reoxygenation. Am J Pathol.

[38] Ait Mou Y (2009). Late exercise training improves non-uniformity of transmural myocardial function in rats with ischaemic heart failure. Cardiovasc Res.

[39] Fenix AM (2018). Muscle-specific stress fibers give rise to sarcomeres in cardiomyocytes. Elife.

[40] Dispersyn GD (2002). Dissociation of cardiomyocyte apoptosis and dedifferentiation in infarct border zones. Eur Heart J.

[41] Ordoño J, et al. Lactate promotes cardiomyocyte dedifferentiation through metabolic reprogramming [preprint]. 10.1101/2020.07.21.213736 Posted on bioRxiv July 21, 2020

[42] Greenberg B (2016). Calcium upregulation by percutaneous administration of gene therapy in patients with cardiac disease (CUPID 2): a randomised, multinational, double-blind, placebo-controlled, phase 2b trial. Lancet.

[43] Waggoner JR, Kranias EG (2005). Role of phospholamban in the pathogenesis of heart failure. Heart Fail Clin.

[44] Frank K, Kranias EG (2000). Phospholamban and cardiac contractility. Ann Med.

[45] Wickenden AD (1998). The role of action potential prolongation and altered intracellular calcium handling in the pathogenesis of heart failure. Cardiovasc Res.

[46] Schipper DA (2017). Chronic myocardial ischemia leads to loss of maximal oxygen consumption and complex I dysfunction. Ann Thorac Surg.

[47] Sharma A (2014). Human induced pluripotent stem cell-derived cardiomyocytes as an in vitro model for coxsackievirus B3-induced myocarditis and antiviral drug screening platform. Circ Res.

[48] Benjamin EJ (2017). Heart disease and stroke statistics-2017 update: a report from the American Heart Association. Circulation.

[49] Lian X (2013). Directed cardiomyocyte differentiation from human pluripotent stem cells by modulating Wnt/β-catenin signaling under fully defined conditions. Nat Protoc.

[50] Tohyama S (2017). Efficient large-scale 2D culture system for human induced pluripotent stem cells and differentiated cardiomyocytes. Stem Cell Reports.

[51] Tohyama S (2016). Glutamine oxidation is indispensable for survival of human pluripotent stem cells. Cell Metab.

[52] Dubois NC (2011). SIRPA is a specific cell-surface marker for isolating cardiomyocytes derived from human pluripotent stem cells. Nat Biotechnol.

[53] Herron TJ (2010). Purkinje cell calcium dysregulation is the cellular mechanism that underlies catecholaminergic polymorphic ventricular tachycardia. Heart Rhythm.

[54] Dedkova EN, Blatter LA (2012). Measuring mitochondrial function in intact cardiac myocytes. J Mol Cell Cardiol.

[55] Mathur A (2000). Evaluation of fluorescent dyes for the detection of mitochondrial membrane potential changes in cultured cardiomyocytes. Cardiovasc Res.

