# Supplementary material for: In vitro model of ischemic heart failure using human induced pluripotent stem cell–derived cardiomyocytes
Source: JCI Insight. 2021 May 24;6(10):e134368. doi: 10.1172/jci.insight.134368 (PMC8262347; doi:10.1172/jci.insight.134368)

Supplemental Figure 1

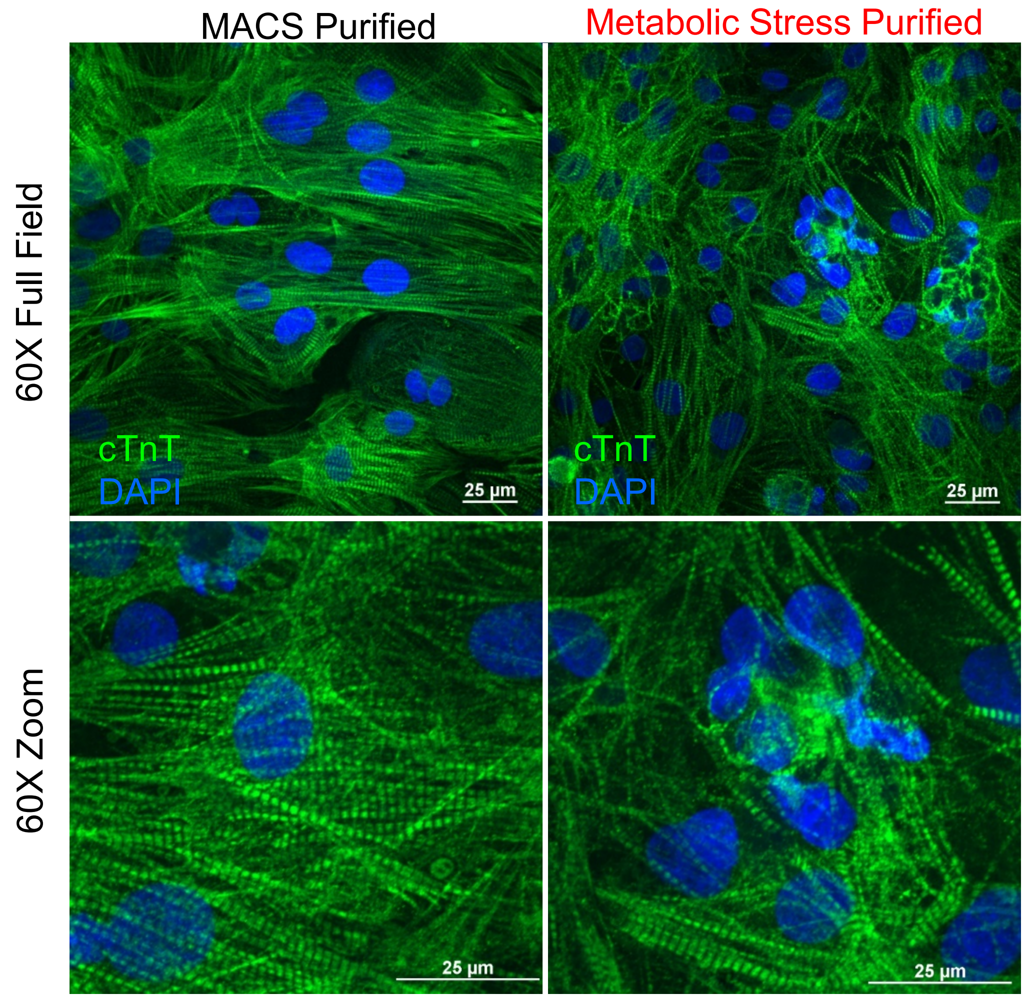

Supplemental Figure 2

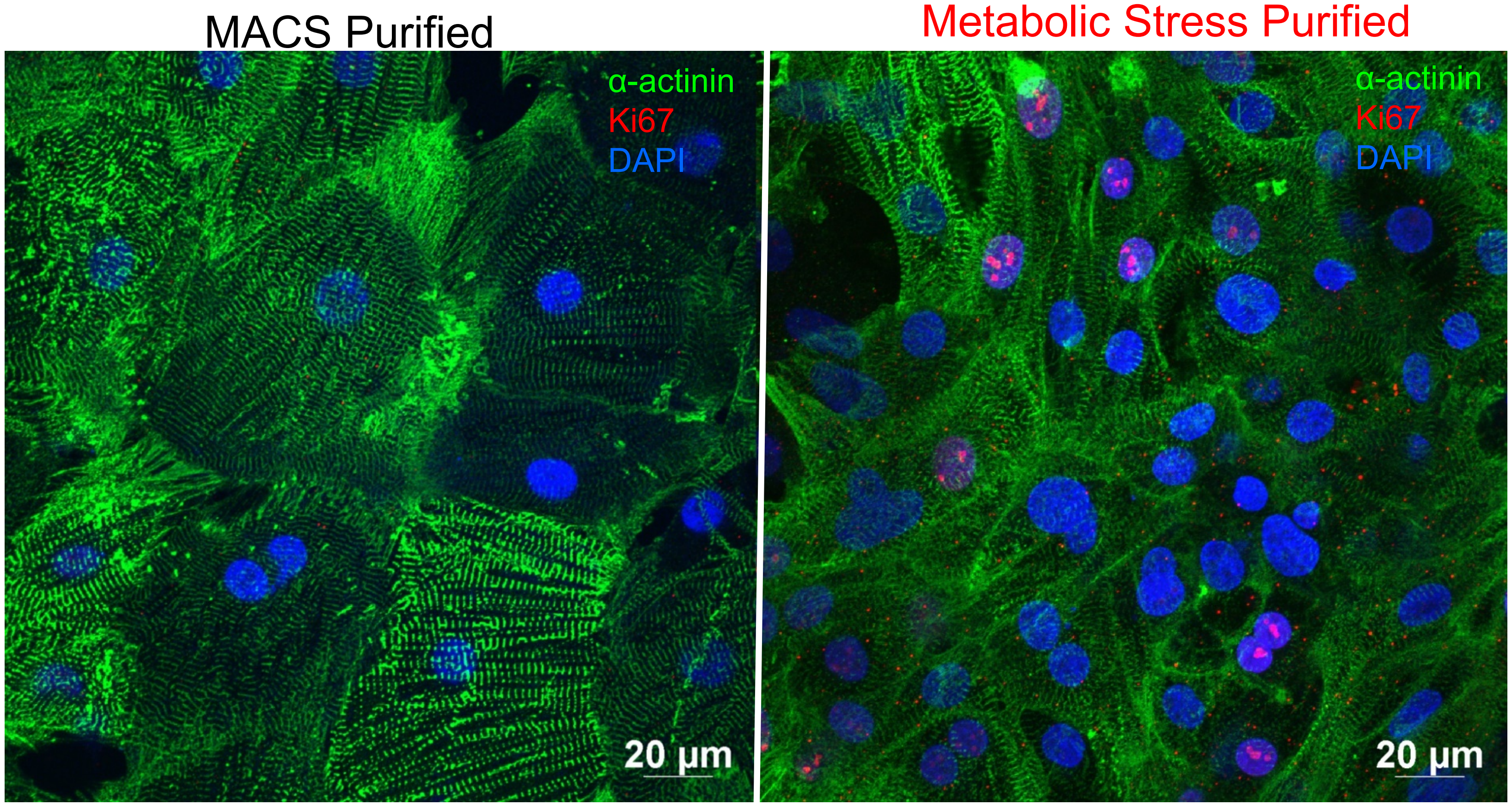

Supplemental Figure 3

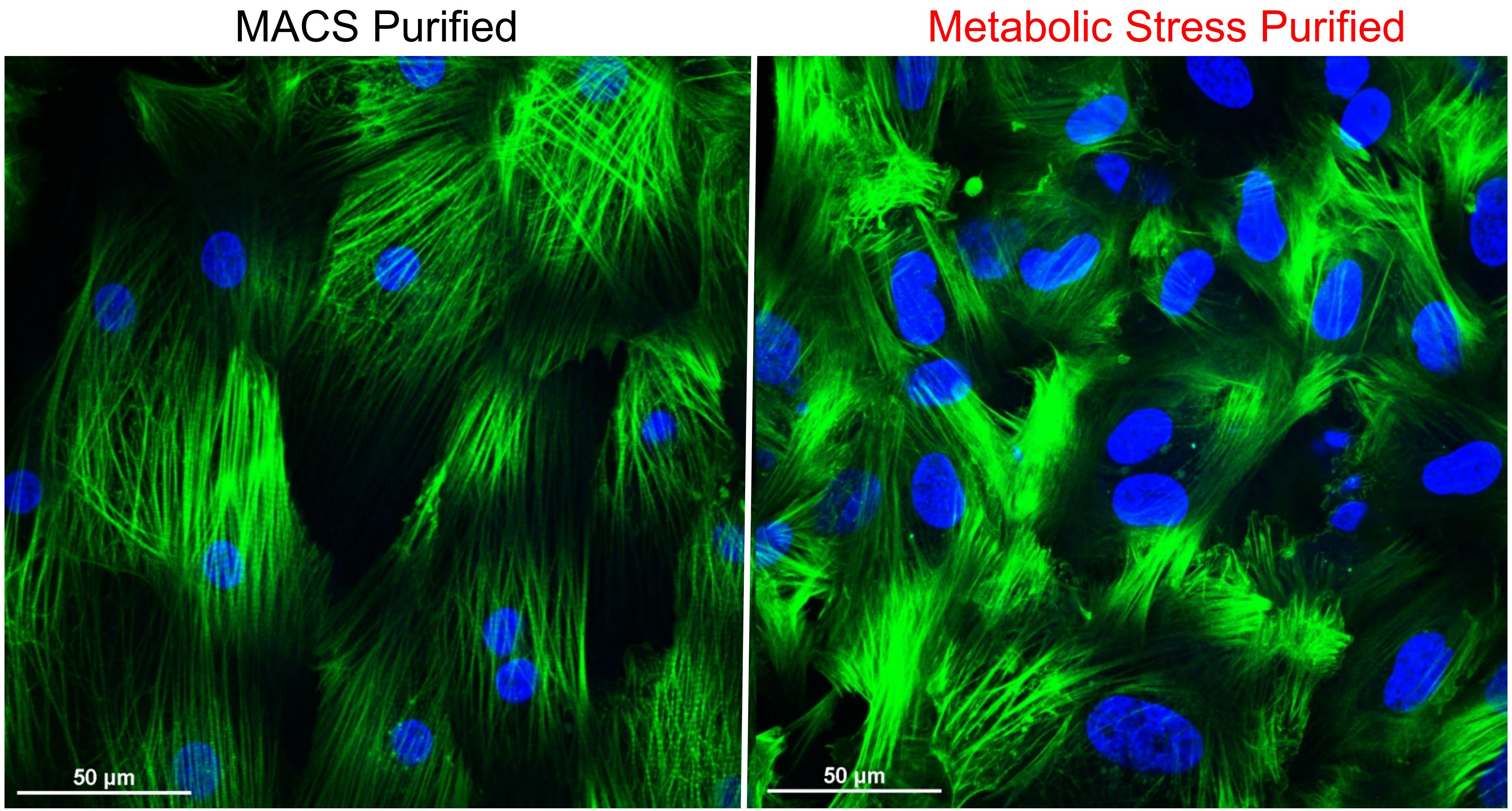

Supplemental Figure 4

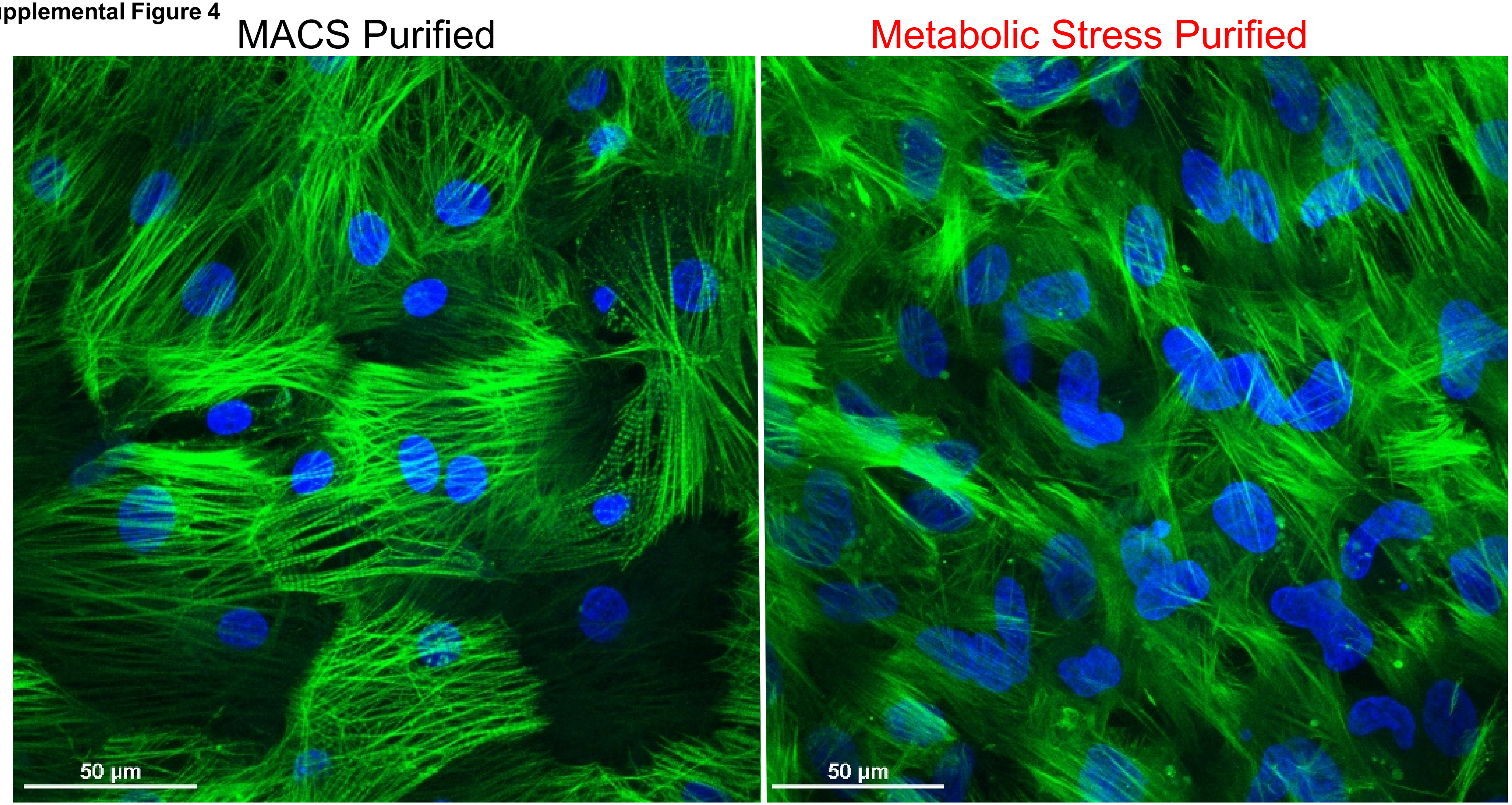

Supplement: Supplemental data [file jciinsight-6-134368-s247.pdf]
